# Mixing and flow-induced nanoprecipitation for morphology control of silk fibroin self-assembly†

**DOI:** 10.1039/d1ra07764c

**Published:** 2022-03-04

**Authors:** Saphia A. L. Matthew, Refaya Rezwan, Jirada Kaewchuchuen, Yvonne Perrie, F. Philipp Seib

**Affiliations:** Strathclyde Institute of Pharmacy and Biomedical Sciences, University of Strathclyde 161 Cathedral Street Glasgow G4 0RE UK philipp.seib@strath.ac.uk +44 (0)141 548 2510; Department of Pharmacy, ASA University Bangladesh 23/3 Bir Uttam A. N. M. Nuruzzaman Sarak Dhaka 1207 Bangladesh; EPSRC Future Manufacturing Research Hub for Continuous Manufacturing and Advanced Crystallisation (CMAC), University of Strathclyde, Technology and Innovation Centre 99 George Street Glasgow G1 1RD UK; Faculty of Nursing, HRH Princess Chulabhorn College of Medical Science, Chulabhorn Royal Academy Bangkok Thailand

## Abstract

Tuning silk fibroin nanoparticle morphology using nanoprecipitation for bottom-up manufacture is an unexplored field that has the potential to improve particle performance characteristics. The aim of this work was to use both semi-batch bulk mixing and micro-mixing to modulate silk nanoparticle morphology by controlling the supersaturation and shear rate during nanoprecipitation. At flow rates where the shear rate was below the critical shear rate for silk, increasing the concentration of silk in both bulk and micro-mixing processes resulted in particle populations of increased sphericity, lower size, and lower polydispersity index. At high flow rates, where the critical shear rate was exceeded, the increased supersaturation with increasing concentration was counteracted by increased rates of shear-induced assembly. The morphology could be tuned from rod-like to spherical assemblies by increasing supersaturation of the high-shear micro-mixing process, thereby supporting a role for fast mixing in the production of narrow-polydispersity silk nanoparticles. This work provides new insight into the effects of shear during nanoprecipitation and provides a framework for scalable manufacture of spherical and rod-like silk nanoparticles.

## Abstract 2

Tuning silk fibroin nanoparticle morphology using nanoprecipitation for bottom-up manufacture is an unexplored field that has the potential to improve particle performance characteristics.

## Graphical abstract

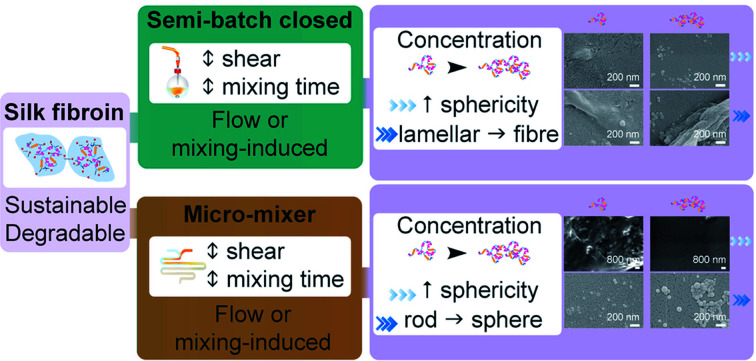

## Introduction

The control of silk fibroin multiscale structure under shear flow is important to the function of this biopolymer in the natural world and can be exploited in organic solvent-induced nanoprecipitation processes. Silk fibroin produced by the *Bombyx mori* silkworm is increasingly proposed for a range of drug delivery applications,^1^ as this biopolymer exhibits several favourable characteristics, including biocompatibility and biodegradability,^2–4^ and a number of products have been translated to the clinic.^5^ A variety of material types^5^ and crystallinities^5–8^ can also be accessed from the reverse-engineered silk solution, as the block copolymer primary structure can exist in a range of polymorphic states, notably silk I–II.^9^ The silk I polymorph has a high composition of β-turns, helices and random coils which bestow aqueous solubility. This metastable solution is found in the silkworm gland or is obtained by regeneration of the degummed silkworm cocoon.^10^

The rate of structural conversion of aqueous silk I to the more thermodynamically stable and solid silk II structure can be increased by displacement of the protein hydration layer with a water-miscible organic solvent^11–13^ and the application of physical shear forces under flow.^10^ The intermolecular hydrogen-bonding ability of silk molecules under shear flow enables the spinning of liquid silk dope at ambient conditions and remarkably little work input.^14^ Similarly, this fundamental property determines the outcome of high shear fluid processing of the regenerated aqueous solutions into structures such as nanoparticles.

The physicochemical properties of nanoparticle drug delivery systems dictate the *in vivo* performance following parenteral administration, including immunogenicity, volume of distribution, controlled drug release and intracellular trafficking.^15^ Nanoparticles smaller than 100 nm in size can be filtered from systemic circulation by the hepatic endothelial fenestration, thereby lowering plasma half-life by accumulation in the liver.^15^ Conversely, the plasma half-life of nanoparticles above 200 nm in size can be reduced due to opsonisation and clearance by the mononuclear reticuloendothelial system.^15^ Therefore, nanoparticles of sizes between 100 and 200 nm with a low polydispersity index have shown the greatest clinical success as drug delivery systems.^15^ The nanoparticle morphology can also impart specific delivery and rheological properties^16,17^ and a change from spherical to cylindrical carriers has been shown to improve circulation *in vivo*.^18^

Silk particles of sub-micron size (25–200 nm) are suitable for drug delivery and can be manufactured by eight major methods (reviewed previously^19^): capillary microdot printing,^20^ desolvation,^11,13,21,22^ supercritical CO_2_,^23^ electrospraying,^24^ emulsification,^25^ ionic liquid dissolution,^4^ milling^26^ and electrogelation.^27^ Of these methods, desolvation is an accessible, low-energy–expenditure nanoprecipitation process which has been used to tune the key quality attributes of protein^12,13,28^ and polymeric^29^ nanoparticles. The one-step fabrication technique involves solvent shifting of aqueous, molecularly dissolved silk using at least a 200% v/v excess of a water-miscible organic solvent^11,21,22^ in which at least one of the blocks has a low solubility, resulting in supersaturation and spontaneous precipitation.^29,30^

Silk nanoformulations have many advantages, but further progress is needed for the manufacture of non-spherical particles by nanoprecipitation and for a better understanding of the silk self-assembly mechanism in semi-batch and continuous formats. Nanoprecipitation occurs due to the interdiffusion of the antisolvent and water molecules; therefore, it is highly dependent on the mixing conditions, as well as on the composition of the aqueous, solute and antisolvent components used in the mixture.^29,30^ This raises concerns regarding the commonly accepted lab-scale process of manual drop-by-drop silk feeding to an organic antisolvent in semi-batch format (*e.g.*^11^ and 31), as this method results in a time-dependent change in the mixture composition. For this reason, in the last decade, microfluidics has emerged as an alternative to dropwise fabrication, as it can provide a continuous route that subjects all solute molecules to the same conditions, thereby allowing increased control over the mixture composition. Microfluidics have been used most extensively for silk water-in-oil emulsions, with narrow polydispersity particles with rod and spherical morphologies achieved by flow-focusing droplet microfluidics (145–200 μm).^32,33^ However, the micro-size of the emulsions obtained using microchannels^32–34^ limits the application of the resulting particles. The low flow rates required for the generation of nano-sized emulsions (51–1500 nm)^25^ can also limit the throughput speed of production on scale-up.

One solution has been the use of the staggered herringbone micromixer, which can provide high-throughput microfluidic-assisted manufacture of nanoparticles (110–310 nm) using silk nanoprecipitation in acetone and isopropanol antisolvents.^12^ High mixing efficiency occurs in the microchannel as the fluid interface is stretched and folded by asymmetric bas-relief herringbone structures that cause chaotic advection by creating continually switching transverse vortices.^35^ The staggered herringbone micromixer therefore decreases the mixing time compared to laminar flow regimes and semi-batch macromixing.^35^ Use of this micromixer has demonstrated a dependence of the physicochemical properties of the nanoparticles on the solvent properties, the total flow rate and the flow rate ratio of the aqueous and organic phases.^12^

An optimal nanoparticle batch, with a size of 110 nm, a polydispersity of 0.14, a zeta potential of −29.8 mV, a spherical morphology and aqueous stability, has been obtained in a staggered herringbone micromixer using a flow ratio of 5 : 1 isopropanol to silk and a total flow rate of 1 mL min^−1^.^12^ These conditions were used in further work to analyse the impact of the precursor molecular weight on nanoparticle properties by varying the sericin degumming time of silk cocoons.^13^ In both manual semi-batch and continuous formats, increasing the degumming time resulted in a greater molecular weight polydispersity of the silk stocks and reduced the nanoparticle size, polydispersity and zeta potential.^13^ By comparison, semi-batch production resulted in a significant decrease in surface charge,^13^ indicating that changes in the mixing time and the supersaturation between the two formats impacted the process of silk–silk association. However, spherical particle morphologies were consistently obtained for regenerated silk stocks.

The aim of the present study was to control the morphology and multiscale structure of silk self-assembly by varying the shear processing and supersaturation conditions under bulk and microfluidic mixing regimes. The effects of mixing on self-assembly were assessed across a range of silk precursor concentrations and shear rates using semi-batch mixing conditions of low and high mixing times as examples of bulk mixing processes for comparison with the staggered herringbone micromixer. This comparative work illustrates that control of silk nanoprecipitation across multiple length scales can be achieved under conditions of high shear and fast mixing and provides a platform for tuning high-shear, semi-batch processes.

## Experimental

### Materials

All studies were carried out at 18–22 °C, unless otherwise stated. Reagents and solvents were acquired from Acros Organics™ or Sigma Aldrich at >98% purity and used without additional purification. Runs for nanoparticle production in continuous format were defined as complete nanoparticle collection using one silk precursor solution (1 mL). Nanoparticle batches were obtained in continuous format and in semi-batch format using 0.5% w/v aqueous silk by mixing the product suspensions from three runs that used the same silk precursor solution prior to ultracentrifugation. Each independent experiment was repeated in triplicate using three different silk precursor stock solutions.

### Regeneration of *B. mori* silk

Silk fibroin from *B. mori* cocoons was regenerated using the 1 h sodium carbonate and 4 h lithium bromide method detailed previously,^36^ and described in video format elsewhere.^11^

### General drop-by-drop manufacture of silk nanoparticles in semi-batch format

**Fig. 1:**
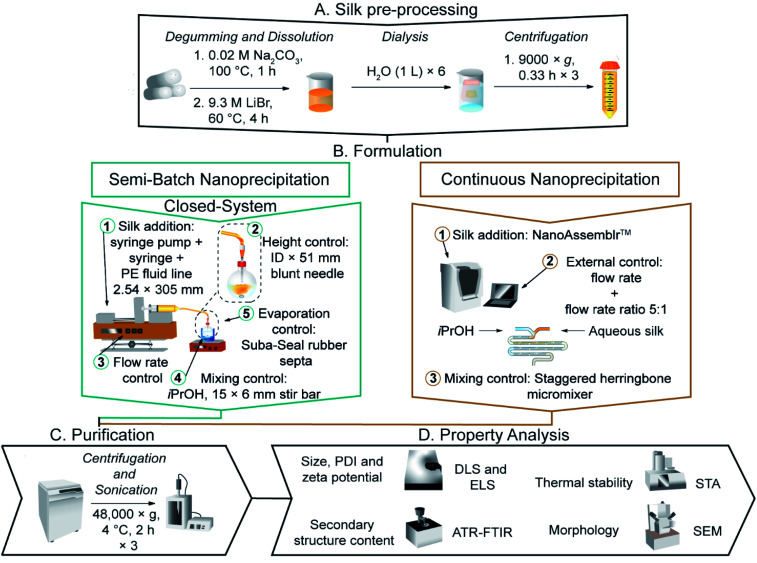
The nanoprecipitation workflow for the preparation and purification of silk nanoparticles *via* desolvation in isopropanol. (a) In semi-batch format, the four formulation processing steps are: (1) loading of a bubble-free aqueous silk solution into a syringe equipped with blunt needle. (2) The silk feed position. (3) The flow rate control of silk solution. (4) Control of mixing time *via* the stirring rate during addition. In continuous format, the three formulation processing steps are: (1) loading of bubble-free aqueous silk and isopropanol into syringes and the NanoAssemblr™ microfluidic chip. (2) The flow rate control of silk solution and the flow rate ratio control of isopropanol: silk. (3) Control of mixing time *via* the micromixer chip design.

Silk nanoparticles were manufactured by the addition of a 3% w/v silk solution (1 mL) to isopropanol (5 mL) in a short-neck round-bottom flask. The silk feed was supplied at room temperature using a syringe pump (Harvard Apparatus 22, Holliston, MA, USA) held at an inclination of 0–0.1° and equipped with a BD PLASTIPACK™ syringe, polyethylene Luer lock fluid line (2.54 × 305 mm) and blunt needle (Fig. 1). The flask was stoppered, and the mother liquor was incubated at room temperature for no longer than 0.5 h before dilution with ultrapure H_2_O in a polypropylene ultracentrifugation tube and centrifugation at 48 400 × *g* for 2 h at 4 °C (Beckmann Coulter Avanti® J-E equipped with JA-20 rotor). The supernatant was removed, the pellet was resuspended in ultrapure H_2_O (20 mL) and the suspension was sonicated twice for 30 seconds at 30% amplitude with a Sonoplus HD 2070 sonicator (ultrasonic homogeniser, Bandelin, Berlin, Germany). Ultrapure H_2_O (23 mL) was added to the suspension, and the centrifugation, washing and resuspension steps were repeated for a total of three times. The final pellet was suspended in ultrapure H_2_O (2–3 mL) and stored at 4 °C until use. Each experiment was repeated in triplicate using three different silk precursor stocks.

Calculations for needle residence time and shear rate were based on the literature value for dynamic viscosity (27 mPa s) of regenerated 3% aqueous silk^37^ and the calculated density (1.02 g mL^−1^) for the 3% w/v aqueous silk solution, with an assumed newtonian flow (Table 1).^37^ The Reynolds number was estimated based on the internal diameter of the needle.^38^ An upper limit of the residence time was estimated using the linear velocity and the needle length.^39^ The maximum shear rate was taken as the wall shear rate and, for simplicity, the shear rate calculations used the geometry of a straight cylinder. Calculations for the 3, 10 and 50 mL syringes used in the study were performed similarly, using the internal diameters stated by the manufacturer (Table S1†).

**Table 1:** The estimated flow characteristics of the needles used in the semi-batch system and the micro-mixer indicated that increasing the flow rate of silk in the feed needle in semi-batch format and of the aqueous silk–isopropanol mixture in the microchannel increased the wall shear rate^a^

Semi-batch format					Micro-mixer		
Needle internal diameter/mm	Flow rate/mL min^−1^	Residence time/ms	Maximum shear rate/s^−1^	Re	Flow rate/mL min^−1^	Maximum shear rate/s^−1^	Re
0.33	0.017	15 335	80	0.04	0.001	80.1	0.04
	1.000	261	4724	2.5			
	3.510	74	16 581	8.5	0.059	4727	2.4
	7.000	37	33 068	17			
	8.485	31	40 083	21			
	16.96	15	80 119	41	0.50	40 073	20
0.41	3.510	115	8646	6.9			
0.6	3.510	246	2759	4.7			
0.84	3.510	481	1005	3.4	1.00	80 114	40
1.19	3.510	966	354	2.4			
1.60	3.510	1746	145	1.8			

a The average wall shear rate under laminar and newtonian flow is reported. The micromixer geometry was simplified to a rectangular channel by ignoring the groove depth. Residence times in the fluid line and needles were estimated using the linear velocity while the volumetric flow rate was used for the micromixer.

### The effects of flow rate and initial addition height in closed, semi-batch format

Silk nanoparticles were manufactured in 10 mL round-bottom flasks using a blunt needle (0.33 × 51 mm). At room temperature, a 3% w/v silk solution was added to isopropanol at flow rates of 0.017, 1.000, 3.510 or 7.000 mL min^−1^, while varying the initial addition height at 0.7, 2.1 or 3.5 cm from the isopropanol surface. The Reynolds numbers at 0.017, 1.000, 3.510 and 7.000 mL min^−1^ (calculated as 5.4 × 10^−3^, 0.32, 1.1 and 2.2 for the fluid line, and as 0.04, 2.5, 8.5 and 17 for the needle) indicated laminar flow (Tables S2† and 1).

#### The effect of needle diameter in the closed, semi-batch format

The 3% w/v silk solution was added at a rate of 3.510 mL min^−1^ to isopropanol from an initial addition height of 3.5 cm from the isopropanol surface. The needle diameter was varied between 0.33, 0.41, 0.60, 0.84, 1.19 and 1.60 mm. The Reynolds numbers for the needle diameters of 0.41, 0.60, 0.84, 1.19 and 1.60 mm were calculated as 6.9, 4.7, 3.4, 2.4 and 1.8, respectively (Table 1).

#### The effect of stirring rate and feed addition height in the closed, semi-batch format

Silk nanoparticles were manufactured using a blunt needle (0.33 × 51 mm). The silk solution flow rate was 1.000 mL min^−1^, the initial addition height was 0.0, 1.75 or 3.5 cm from the isopropanol surface, and the stirring rate, provided by an egg-shaped stir bar (15 × 6 mm), was 0, 200, 400 or 800 rpm. The Reynolds number of the stirred vessel, estimated using a cylindrical geometry as ≈1300, 2500 and 5030 at stirring rates of 200, 400 and 800 rpm, respectively (Table S3†), indicated turbulent flow within the vessel at 400 and 800 rpm.

### The effect of flow rate and concentration in the closed, semi-batch format

Silk nanoparticles were manufactured using a blunt needle (0.33 × 51 mm). Silk solution was added to isopropanol at rates of 0.017, 1.000, 8.485 or 16.96 mL min^−1^ from an addition height of 1.75 cm and the stirring rate was varied from 0 to 400 rpm using the egg-shaped stir bar. The silk concentration was 0.5, 2 or 3% w/v. The Reynolds numbers at 8.485 and 16.96 mL min^−1^ (calculated as 2.7 and 5.4 for the fluid line and 21 and 41 for the needle) indicated laminar flow (Table 1).

#### Dual indicator system for mixing time in the semi-batch format

The rotational speed investigated at the 5 mL scale ranged from 200–800 rpm in increments of 200 rpm, and the initial addition height was 0, 1.75 or 3.5 cm. The round bottom flask was submerged in water in a clear acrylic box (10.3 × 10.3 × 5 cm) to reduce surface reflections. An LED panel (RALENO, Seattle, WA, USA) was fixed at the back of the stirring plate to provide constant illumination at 5600 K colour temperature and at 100% brightness.

The dual indicator system for mixing time described by Melton *et al.*^40^ and Weheliye *et al.*^41,42^ was used, with some adaptations. Stock solutions of thymol blue (0.095 mg mL^−1^) and methyl red (0.135 mg mL^−1^) were prepared in ethanol. The working solution was prepared by mixing and diluting the stock solutions to give 4.3 mg mL^−1^ thymol blue and methyl red in 70% v/v ethanol. To each 5 mL aliquot of the working solution, 0.5 M HCl was added at 0.5 mL L^−1^, and the system was equilibrated for at least 10 revolutions. An equivalent amount of NaOH (10.5 μL of 0.15 M NaOH) was then added to the mixture using a 20 μL Eppendorf pipette (attached to a clamp stand for control of the feed location and height). The mixing process was captured on an iPhone SE (Apple, Cupertino, CA, USA) reverse camera at a capture speed and resolution of 240 fps and 1080 p using FiLMiC Pro (FiLMiC Inc., Seattle, WA, USA). Each condition was repeated at least four times.

Images were extracted at 240 fps using FFmpeg.^43^ The images were processed using custom MATLAB (Mathworks, Natick, USA) scripts to apply rectangular masks of 18 000 pixels and to calculate the standard deviation of the normalised green channel intensity, as described by Rodriguez *et al.*^44^ The standard deviation of the fully mixed condition was calculated as the average of the final ten images, and the mixing time (*t*_95%_) was estimated as the time required to reach 95% of the standard deviation at the fully mixed condition (Table 2).

**Table 2:** The estimated mixing characteristics of the semi-batch reactor and the micromixer indicated that increasing the feed height and stirring rate in semi-batch format decreased the bulk mixing time and increasing the flow rate in microfluidic format reduced the mixing time

Semi-batch reactor			Micro-mixer		
Feed height/cm	Stirring rate/rpm	Mixing time/s	Flow rate/mL min^−1^	Residence time/ms	Mixing time/ms
0	200	16 ± 5.1	0.001	120 000	14 500
	400	15.7 ± 3.7			
	800	5.3 ± 1.1	0.059	2034	306
1.75	200	28.0 ± 3.7			
	400	8.4 ± 3.0	0.50	240	40
	800	2.0 ± 1.0			
3.50	200	16.9 ± 3.5	1.00	120	21
	400	3.3 ± 3.0			
	800	0.7 ± 0.1			

### Semi-batch droplet analysis

#### Volume and time of flight

The semi-batch system was used to determine the average volume of a droplet at flow rates of 0.017, 1.000, 3.510, 7.000, 8.485 and 16.96 mL min^−1^. During the time taken to extrude a total volume of 1 mL, the number of droplets was captured on an iPhone SE (Apple, Cupertino, CA, USA) reverse camera at a capture speed of 30 fps at 0.017 mL min^−1^ and 240 fps for flow rates greater than 0.017 mL min^−1^. The resolution was kept constant at 1080 p using FiLMiC Pro (FiLMiC Inc., Seattle, WA, USA). The experiments were repeated in triplicate.

#### Fluid velocity, droplet diameter and diffusion scales

**Fig. 2:**
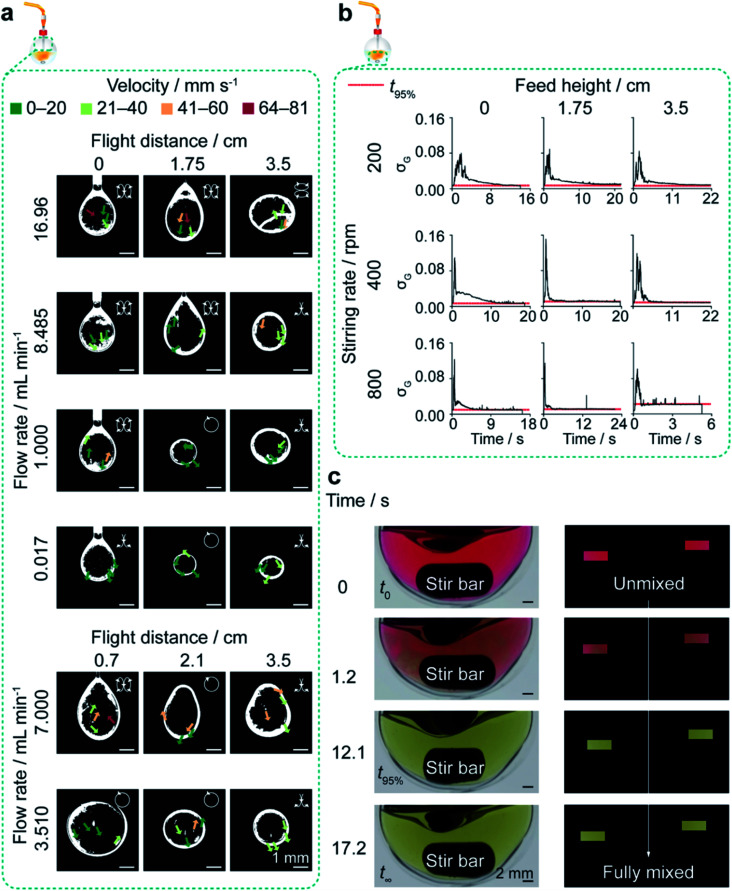
Exemplary characterisation of flow and mixing properties in the closed semi-batch system. Circulatory flow in silk droplets produced at all flow rates and feed heights was observed using silk doped with iron oxide nanoparticles. The mixing time in the reactor decreased as stirring rate and feed height increased and was measured using the colour change of a methyl red and thymol blue mixture from acidic pH (red) to neutral pH (yellow). (a) Processed binary images of the circulatory flow field of droplets extruded from the closed semi-batch system. Insets show the two-dimensional direction of flow. (b) The variation of standard deviation of the normalized green channel (*σ*_G_) across scale in the open semi-batch system and across feed height and stirring rate in the closed semi-batch system. (c) The raw images and processed masks showing the colour evolution within the flask at the optimal stirring rate of 400 rpm and feed height of 1.75 cm.

The closed semi-batch system was used to determine the flow rate within droplets extruded at flow rates of 0.017, 1.000, 3.510, 7.000, 8.485, and 16.96 mL min^−1^ at relevant heights between 0 and 3.5 cm (Fig. 2a). At least three droplets of 3% w/v silk solution with 0.3% w/w iron(iii) oxide (synthetic spherical particle with 99.995% < 325 mesh (∼45 μm) size, >96.8% purity, 4.6 g cm^−3^ solid density and 0.8–1.2 g cm^−3^ bulk density from Inoxia Ltd, Sweden) were imaged on an iPhone SE (Apple, Cupertino, CA, USA) reverse camera equipped with 15× macro lens (Shenzhen Apexel Technology Co., Guangdong, Shenzhen, China) at a focal length of 1.5 cm. The resolution was kept constant at 1080 p using FiLMiC Pro (FiLMiC Inc., Seattle, WA, USA) and images were extracted at 240 fps using FFmpeg.^43^ The images were processed using custom MATLAB (Mathworks, Natick, USA) scripts to perform greyscale conversion, contrast-limited adaptive histogram equalization and binary image conversion based on luminance. The particle velocities were measured by manual tracking using ImageJ v1.52n (National Institutes of Health, Bethesda, MD, USA). An LED panel (RALENO, Seattle, WA, USA) was fixed behind the droplets to provide constant illumination at 5600 K colour temperature and at 80% brightness. The droplet diameters of at least three droplets were imaged on a Photron FASTCAM SA 1.1 Model 675 K M1 (Photron, San Diego, CA, US) at 3× magnification and measured using Photron FASTCAM Viewer (Photron, San Diego, CA, US). The diffusion length and time scales were calculated assuming fickian diffusion and a silk diffusion coefficient of 2.45 × 10^5^ cm^2^ s^−1^ (Table S4†).^45^

### Microfluidic-assisted manufacture of silk nanoparticles

Silk nanoparticles were manufactured using the NanoAssemblr™ benchtop instrument version 1.5 (model number NA-1.5-16; NanoAssemblr™, Precision Nano-Systems Inc. Vancouver, Canada) equipped with a cyclic olefin copolymer microfluidic cartridge (product codes: 1207 and 1151-034 BENCHTOP CARTRIDGE), as described elsewhere.^12^ Using the Y-junction of the two 25 mm inlet channels, the fluids were mixed in a 27 mm rectangular mixing channel (79 μm × 200 μm) having a series of raised grooves (31 μm × 50 μm) and four switchback turns.^46^ A 1 mL volume of aqueous silk solution (0.5–3% w/v) and isopropanol (5 mL) were injected into separate chamber inlets, and the nanoparticles formed in the staggered herringbone mixer were collected from the outlet. The total flow rate of the isopropanol and silk mixture was varied between 0.001–1.0 mL min^−1^, and the flow rate ratio was 5 : 1. The cartridge was cleaned between runs using a water wash and a prime. The water wash consisted of a flow ratio of 1 : 1 ultrapure H_2_O/ultrapure H_2_O, a total volume of 2 mL and a total flow rate of 4 mL min^−1^; the wash was repeated in triplicate. The priming procedure consisted of a flow ratio of 5 : 1 isopropanol/ultrapure H_2_O, a total volume of 6 mL and a total flow rate of 1 mL min^−1^. The mother liquor suspension was incubated for no longer than 0.5 h before purification.

Calculations were based on the literature viscosity value (3.14 mPa s) and density (0.837 g mL^−1^) values for the 5 : 1 v/v isopropanol/water mixture measured at 20 °C,^47^ and newtonian flow was assumed.^37^ Increasing the flow rate from 0.001 to 1 mL min^−1^ resulted in mixing time estimates of 21 to 14 500 ms respectively, according to the manufacturer's guidelines and based on an analytical model for a similar system published elsewhere, by calculating the Peclet number to achieve a coefficient of variation of <0.1 (Table 2).^48^ The Peclet numbers were estimated as 4.27 × 10^8^, 2.52 × 10^10^, 2.14 × 10^11^, and 4.27 × 10^11^ using the hydraulic diameter of the channel^38^ (142 μm) and the diffusion coefficient (3.5 × 10^−10^ m^2^ s^−1^) of the 5 : 1 isopropanol/water mixture.^49^ The Reynolds numbers (0.04, 2.4, 20, 40) indicated laminar flow (Table 1). An upper limit of the residence time was estimated using the total fluidic volume and flow rate.^39^ The residence time was longer than the mixing time at all flow rates, indicating that complete mixing had occurred in the micromixer. The maximum shear rate was taken as the wall shear rate, with the assumptions that chaotic advection did not significantly affect the wall shear rate and that it created a significantly lower shear within the channel. For simplicity, the shear rate calculations used the geometry of a straight rectangular channel and did not take into account the groove depth.^50^

#### Yield of silk nanoparticles

The total mass of silk nanoparticles was recorded by transferring 450 μL of the suspension to a pre-weighed microcentrifuge tube and recording the total mass, followed by freezing at −80 °C for 5 h and freeze-drying (Christ Epsilon 1-4, Martin Christ Gefriertrocknungsanlagen GmbH, Osterode, Germany) for 24 h at −10 °C and 0.14 mbar. The dry mass was then recorded and the yield calculated as detailed previously.^36^ This process was repeated twice, and an average yield was reported. Freeze-dried silk nanoparticles were stored in a vacuum desiccator at 25 °C until use.

#### Physicochemical characterisation of the silk nanoparticles

The silk nanoparticle size (*Z*-average of the hydrodynamic diameter), polydispersity index and zeta potential were measured in triplicate in ultrapure H_2_O at 25 °C by dynamic light scattering (DLS) (Zetasizer Nano-ZS Malvern Instrument, Worcestershire, UK). Nanoparticle suspensions were prepared for measurement by vortex application for 20 seconds and sonication twice at 30% amplitude for 30 seconds, unless otherwise stated. Refractive indices of 1.33 and 1.60 were used for H_2_O and protein, respectively.

#### Secondary structure measurements of silk nanoparticles

Positive silk II controls consisted of autoclaved silk films and silk films treated with 70% v/v ethanol/ultrapure H_2_O. Positive silk I controls were air-dried silk films and freeze-dried silk. The air-dried silk films (0.3 mL) were drop-casted from fluid handling systems following extrusion at a height of 3.5 cm using varying silk concentrations (0.5–3.0% w/v), flow rates (0.017–16.96 mL min^−1^) and needle diameters (0.33–1.60 mm). The secondary structures of silk films, freeze-dried powders and freeze-dried nanoparticles were analysed by Fourier transform infrared spectroscopy (FTIR) on an ATR-equipped TENSOR II FTIR spectrometer (Bruker Optik GmbH, Ettlingen, Germany). Each FTIR measurement was recorded in absorption mode over the wavenumber range of 400 to 4000 cm^−1^ at 4 cm^−1^ resolution for 128 scans and then corrected for atmospheric absorption using Opus (Bruker Optik GmbH, Ettlingen, Germany). The second derivatives of the background-corrected FTIR absorption spectra were analysed in OriginLab 19b® (Northampton, Massachusetts, USA) by adapting a literature protocol.^51^ Each second derivative was smoothed twice using a seven-point Savitzky–Golay function with a polynomial order of 2. The amide I region was analysed by interpolation of a non-zero linear baseline between 2–3 of the highest values in the 1600–1700 cm^−1^ range. Peak positions were identified by applying the second derivative, and peaks were fitted in the amide I region using non-linear least squares with a series of Gaussian curves (Fig. S1†). The position, width and height of each peak were allowed to vary, while the peak area could take any value ≤0. Deconvoluted spectra were then area-normalised, and the relative area of each band was used to calculate the secondary structure content according to literature band assignments.^33,52^

The correlation coefficients I of silk films, freeze-dried silk and nanoparticles were calculated by adaptation of a literature protocol^53^ using the air-dried silk film of an aqueous silk precursor batch as a reference. The second derivative of the FTIR absorption spectra was calculated and smoothed twice with a five-point Savitzky–Golay function and a polynomial order of 2. The processed silk sample was then compared with the reference over the spectral range 1600–1700 cm^−1^ using eqn (1).1
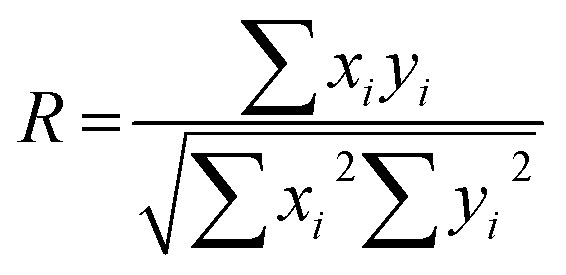
where *x*_*i*_ and *y*_*i*_ are the derivative values of the air-dried silk film and processed silk sample at the frequency *i*.

#### Scanning electron microscopy of silk nanoparticles

A 1 mg mL^−1^ silk nanoparticle suspension (10–20 μL) was pipetted onto a silicon wafer and lyophilised at −10 °C and 0.14 mbar for 24 h. The wafer was sputter-coated with gold (15 nm) using a low vacuum sputter coater (Agar Scientific Ltd, Essex, UK, and ACE200, Leica Microsystems, Wetzlar, Germany) and imaged with an FE-SEM SU6600 instrument (Hitachi High Technologies, Krefeld, Germany) at 5 kV and 40k magnification or an FEI Quanta 250 FEG-ESEM instrument (Oxford Instruments, Abingdon, U.K) at 5 kV and 10, 20 and 40k magnification. The images were processed using ImageJ v1.52n (National Institutes of Health, Bethesda, MD, USA), Adobe Lightroom and Abode Illustrator (Adobe, San Jose, CA, USA).

#### Statistical analyses

Data were analysed using Microsoft® Excel® 2019 (Microsoft Office 365 ProPlus Software, Redmond, WA, USA), Minitab® (Minitab® Statistical Software, State College, PA, USA) and GraphPad Prism 8.2.1 (GraphPad Software, La Jolla, CA, USA). Normality of the data distributions was assumed throughout. The equivalence of variance for sample pairs and multiple groups was identified with the *F*-test and Bartlett's test. Sample pairs were analysed using the independent *t*-test. Multiple groups across one independent variable were evaluated by one-way analysis of variance (ANOVA), followed by Tukey's pairwise multiple comparison *post hoc* test, or by the Brown–Forsythe and Welch ANOVA tests, followed by the Dunnett T3 pairwise multiple comparison *post hoc* test. Two-way ANOVA was used to compare multiple groups across two independent variables, followed by Tukey's pairwise multiple comparison post-hoc test when a significant interaction was shown and by Tukey's main and simple effect multiple comparison *post hoc* tests when no interaction was shown. Statistical significance, identified using *post hoc* tests, was as follows: *p < 0.05, **p < 0.01, ***p < 0.001, ****p < 0.0001. All data are displayed as the mean ± standard deviation, with the number of experimental repeats (*n*) shown in each figure legend.

## Results

### Silk nanoparticle characterisation

#### The effect of flow rate, addition height and stirring rate in closed semi-batch format

In the absence of stirring, increasing the flow rate of the silk solution from 0.017 mL min^−1^ (mixing-induced nucleation) to 7.000 mL min^−1^ (shear-induced nucleation), while also increasing the silk feed addition height, resulted in large, polydisperse nanoparticles regardless of factor levels (Fig. S2a†). Although the nanoparticles remained large and polydisperse scanning electron microscopy (SEM) indicated that moving from shear-induced to mixing-induced nucleation by increasing the needle diameter reduced the extent of self-assembly (Fig. S2c and d†).

**Fig. 3:**
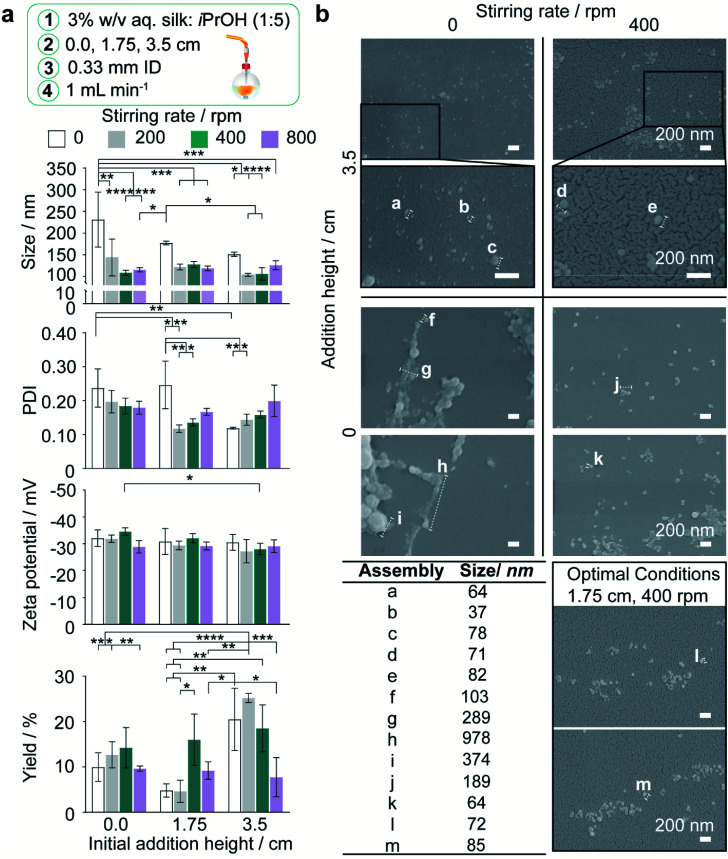
Impact of feed height and stirring rate on nanoprecipitation of 3% w/v aqueous silk in the semi-batch closed-system at 1 mL min^−1^ flow rate. (a) Hydrodynamic diameter, polydispersity index (PDI), zeta potential and yield of silk nanoparticles. Two-way ANOVA was used to compare multiple groups across feed height and stirring rate, followed by Tukey's pairwise multiple comparison *post hoc* test for size, polydispersity index and yield and Tukey's simple effect multiple comparison *post hoc* test for zeta potential. (b) Scanning electron microscopy showed that lower curvature morphologies were obtained as stirring rate and addition height decreased, due to an increasing degree of secondary self-assembly. Error bars are hidden in the bars and plot symbols when not visible, ±SD, *n* = 3. Asterisks denote statistical significance determined using *post hoc* tests as follows: *p < 0.05, **p < 0.01, ***p < 0.001, ****p < 0.0001. Scale bars 200 nm.

As manufacture in the closed, semi-batch system without stirring resulted in large nanoparticles with a wide size distribution, the effect of varying the time and shear stress of mixing was then determined at a 1.000 mL min^−1^ flow rate (shear-induced nucleation). The mixing time was reduced by increasing the stirring rate from 0 to 800 rpm and increasing the droplet velocity by raising the feed height from 0.0 cm to 3.5 cm (Tables 2, S4,† and Fig. 2). Increasing the levels of both these factors significantly reduced the nanoparticle size and polydispersity index (Fig. 3a). At 0 rpm, an increase in the feed height from 0.0 to 3.5 cm significantly decreased the nanoparticle size (from 232 to 151 nm) and the polydispersity index (from 0.24 to 0.12) (Fig. 3a). Equally, an increase in the stirring rate from 0 to 800 rpm significantly decreased the nanoparticle size to 116 nm at 0.0 cm feed height and the polydispersity index from 0.25 to 0.14 at 1.75 cm feed height. The SEM views confirmed the reduction in assembly size and the increase in nanoparticle curvature as mixing time decreased (Fig. 3b).

Generally, the nanoparticle size and size distribution were optimal at 1.75 and 3.5 cm feed heights combined with stirring rates of 200 and 400 rpm (104–128 nm; 0.12–0.16) (Fig. 3a). As feed height increased, silk nanoparticles were also produced with a higher zeta potential at 400 rpm, and in greater yield at low stirring rates, where the yield reached a maximum of 25% at 3.5 cm and 200 rpm. Further reductions in the mixing time at 3.5 cm (Table 2) obtained by increasing the stirring rate to 800 rpm caused a significant drop in nanoparticle yield to 8%. Overall, the nanoparticle size, polydispersity index and zeta potential were optimised at 400 rpm stirring rate. At 1.75 cm feed height a sufficient yield was returned while maintaining low diffusion length scales and shear exposure caused by circulatory flow during droplet flight (Fig. 2a, 3a, and Table S4†). These factor levels were set for further investigations of the effect of flow rate and silk concentration.

#### The effect of flow rate and silk concentration in closed semi-batch and microfluidic formats

**Fig. 4:**
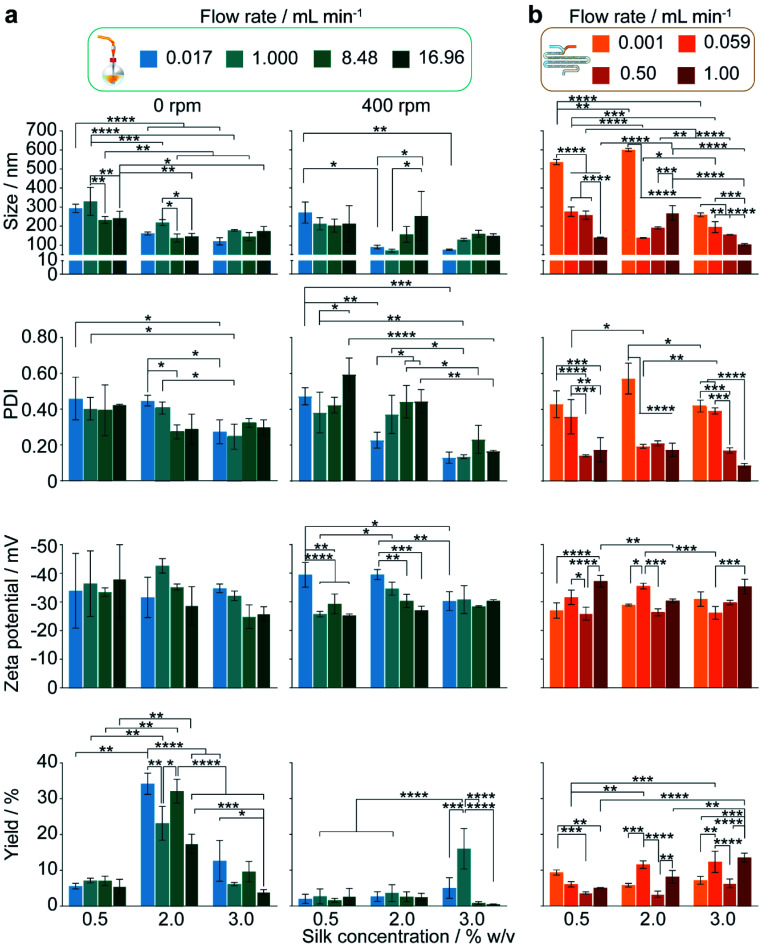
The impact of increasing the flow rate and silk feed concentration in the staggered herringbone micromixer and in semi-batch systems of high and low mixing time. The hydrodynamic diameter, polydispersity index, zeta potential and yield for (a) semi-batch format and (b) microfluidic format. For the unstirred semi-batch processes, two-way ANOVA was used to compare multiple groups across concentration and flow rate, followed by Tukey's pairwise multiple comparison *post hoc* test for yield and Tukey's simple effect multiple comparison *post hoc* test for size, polydispersity index and zeta potential. In stirred semi-batch format and microfluidic format, multiple groups were evaluated by two-way ANOVA, followed by Tukey's pairwise multiple comparison post-hoc test for all properties error bars are hidden in the bars when not visible, ±SD, *n* = 3. Asterisks denote statistical significance determined using *post hoc* tests as follows: *p < 0.05, **p < 0.01, ***p < 0.001, ****p < 0.0001. For ease, all statistically significant interactions have been omitted and are shown in Fig. S3.†

**Fig. 5:**
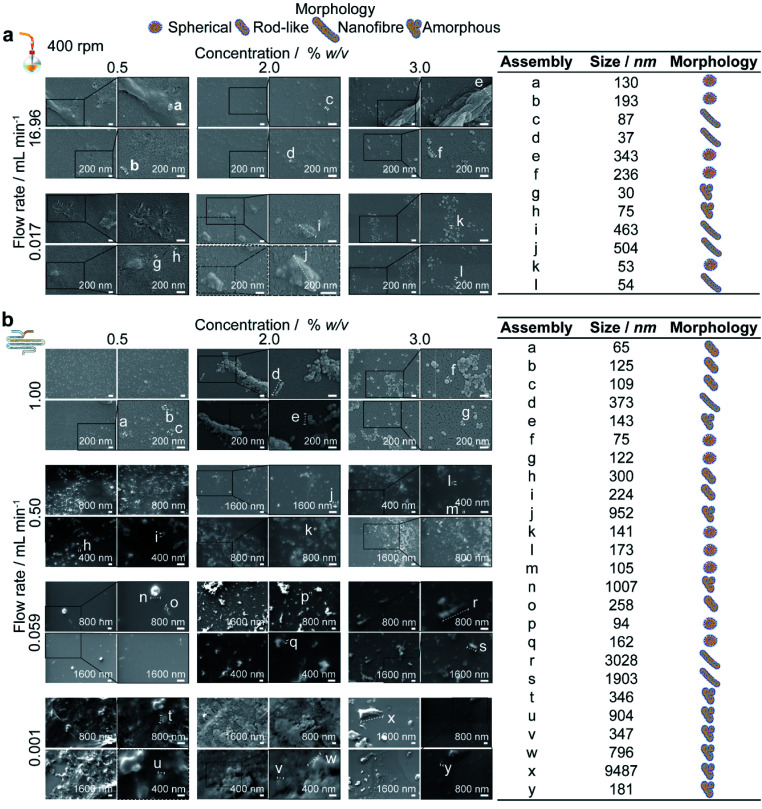
Scanning electron microscopy supported the DLS results and confirmed that for low shear processes in the (a) semi-batch format and (b) microfluidic format, the extent of self-assembly varied inversely with silk concentration while for high shear processes, the extent of assembly was maximised at the 2% silk concentration. Due to an extended growth phase associated with 0.5% silk feeds, rod-like silk nanoparticle morphologies were produced in the micromixer between 0.059–1.0 mL min^−1^, and the particle polydispersity decreased with increasing flow rate.

Under conditions of mixing-induced nanoprecipitation in semi-batch format at a 0.017 mL min^−1^ flow rate and 400 rpm stirring rate, increasing the silk concentration from 0.5 to 2 and 3% significantly decreased the nanoparticle size (from 271 to 89 and 75 nm), polydispersity index (from 0.47 to 0.23 and 0.13) and surface charge (from −39 and 40 to −30 mV) (Fig. 4a and S3a†). Significant decreases in the nanoparticle size (from 294 to 161 and 120 nm) and polydispersity index (from 0.46 to 0.41 and 0.24) also occurred with a concentration increase from 0.5 to 2 and 3% when operating without stirring. The SEM observations revealed an increase in particle sphericity with increasing concentration, from nanofibers and amorphous aggregates formed with 0.5% silk to spherical particles with a high polydispersity at 2% and a narrower size distribution at 3% (Fig. 5a).

Conversely, at the flow rate of 0.001 mL min^−1^ in the micromixer, increases in the silk concentration from 0.5 to 2 and 3% resulted in a significant increase, followed by a decrease, in nanoparticle size (from 536 to 600 to 259 nm), whereas the polydispersity index remained high (ranging from 0.57 to 0.42) (Fig. 4b). Increasing the concentration from 0.5 to 3% did not significantly affect the zeta potential or yield, which ranged from −27 to −31 mV and from 9 to 6%, respectively. The DLS results were supported by SEM observations, which showed a mixture of spherical secondary building units and amorphous aggregates for all silk feeds (Fig. 5b).

At the 400 rpm stirring rate, moving from low to high shear by increasing the flow rate from 0.017 to 16.96 mL min^−1^ caused a significant increase in the nanoparticle size from 89 to 252 nm with 2% silk. The size distribution and zeta potential also significantly increased with flow rate at silk concentrations of 0.5 and 2%. SEM examinations confirmed that these results were due to a greater degree of shear-induced self-assembly for all silk feeds as the flow rate increased (Fig. 5a). For example, at 3% silk concentration, tertiary units were present as nanofibres, while at 0.5% and 2%, amorphous, lamellar aggregates also formed. As for mixing-induced nanoprecipitation, the polydispersity index decreased significantly with increasing silk concentration from 0.5 and 2% to 3% across all high-shear flow rates. The yield increased significantly with silk concentration at a flow rate of 1.000 mL min^−1^ and reached a maximum at 3% silk (tested from 1 to 16%).

Similarly, for the high-shear microfluidic format at flow rates between 0.059 and 1.0 mL min^−1^, increasing the silk concentration from 0.5% and 2% to 3% generally resulted in a significant decrease in particle size and an increase in yield (Fig. 4b). At a flow rate of 0.059 mL min^−1^, the size and polydispersity index significantly decreased (from 276 to 138 nm and from 0.36 to 0.19) with increased silk concentration from 0.5 to 2%. The size, polydispersity index and zeta potential magnitude subsequently decreased (from 138 to 194 nm, 0.19 to 0.39 and −36 to −26 mV) as the silk concentration increased further to 3%. The SEM observations (Fig. 5b) reinforced the DLS results and indicated that the primary assemblies formed using 2 and 3% silk stocks underwent secondary and tertiary assembly to give mixtures containing nanofibre aggregates, whereas the 0.5% silk stock formed rod-like particles in mixtures with amorphous aggregates. At 1.0 mL min^−1^, the opposite trend was observed with an increase in the silk concentration from 0.5 to 2%, as the size and zeta potential significantly increased (from 139 to 269 nm and −37 to −30 mV) due to the increased formation of amorphous aggregates. Subsequent increases from 2 to 3% silk resulted in a decreased nanoparticle size (to 103 nm) and an increased sphericity.

For 0.5 and 3% silk feeds, increasing the flow rate from 0.001 to 1.0 mL min^−1^ caused significant reductions in the assembly size (from 536 to 139 nm and from 259 to 103 nm, respectively) and polydispersity index (from 0.43 to 0.17 and from 0.42 to 0.09, respectively). This size reduction was supported by the observed reduction in aggregates as the flow rate increased for both silk feeds (Fig. 5b). Increasing the flow for 3% silk feeds significantly decreased the zeta potential, from −31 mV at 0.059 mL min^−1^ to −35 mV at 1.0 mL min^−1^. For 0.5% silk, changing the flow rate from 0.001 to 1.0 mL min^−1^ also significantly reduced the zeta potential (from −27 to −37 mV) while for 2% silk feedstocks, increasing the flow rate from 0.001 to 0.059 mL min^−1^ reduced particle size (from 600 to 138 nm), polydispersity index (from 0.57 to 0.19) and zeta potential (from −29 to −36 mV) and increased the yield (from 6 to 12%). These changes were accompanied by a morphological shift from amorphous aggregates at 0.001 mL min^−1^ to spherical nanoparticles and nanofibre agglomerates at 0.059 mL min^−1^. Raising the flow rate to 0.5 and 1.0 mL min^−1^ increased the particle size (to 269 nm) and zeta potential (to −26 mV) while lowering the yield (to 8%). At these flow rates, amorphous and nanofibre aggregates were produced in mixtures with spherical nanoparticles. The SEM views reinforced the DLS measurements and confirmed that, at 0.5 and 1.0 mL min^−1^, increasing the silk feed concentration resulted in a shift from rod-like particles to aggregates and then spherical particles (Fig. 4b).

### Secondary structure measurement

The correlation of the silk nanoparticle secondary structure content with formulation conditions and the shear-induced assembly of the silk precursor by extrusion through the feed needle were evaluated by attenuated total reflectance-FTIR (ATR-FTIR) analysis and deconvolution of the amide I region (Fig. S1†). The structural changes caused by the nanoprecipitation process were also evaluated by analysing the amide I region (1600–1700 cm^−1^) using the spectral correlation coefficient method.^53^

#### The effect of flow rate, addition height and stirring rate in closed, semi-batch format

In the unstirred closed system, increasing the feed rate from 0.017 to 7.000 mL min^−1^ at a 3.5 cm feed height caused a decrease in the β-sheet content of silk nanoparticles from 57% to 52% (Fig. S4a†). Conversely, for the extruded silk, increasing the flow rate from 0.017 to 3.510 mL min^−1^ and 7.000 mL min^−1^ decreased the correlation coefficient from 0.78 to 0.44 and 0.34, respectively (Fig. S4b†). Further, increasing the feed height from 0.7 to 2.1 cm at a feed rate of 7.000 mL min^−1^ decreased the nanoparticle correlation coefficient from 0.47 to 0.32 (Fig. S4a†). The needle diameter had no significant effect on the nanoparticle secondary structure content (Fig. S4c†); however, reducing the needle diameter from 1.60 to 0.33 mm at 7.000 mL min^−1^ increased the β-sheet content of drop-casted films from 25% to 33% (Fig. S4b†). This increase in β-sheet crystallinity of the extruded silk with decreasing needle diameter was reinforced by the reduction in the correlation coefficient at feed rates of 3.510 and 7.000 mL min^−1^ (Fig. S4b†).

In the stirred system, the nanoparticle β-sheet content generally increased with feed height from 0.0 to 1.75 cm (Fig. S4d†). For example, at 200 rpm, increasing the feed height from 0.0 to 1.75 cm increased the β-sheet content from 51% to 56% (Fig. S4d†). Increasing the feed height also caused a main effect decrease in the nanoparticle β-turn content. As the stirring rate increased from 0 to 200 rpm and then to 800 rpm, the nanoparticle antiparallel β-sheet content showed a main effect decrease and then an increase, respectively (Fig. S4d†).

#### The effect of flow rate and silk concentration in closed semi-batch format

For the stirred semi-batch process, the nanoparticle β-sheet content and correlation coefficients ranged between 51–58% and 0.23–0.48, respectively, and did not vary significantly with silk concentration or flow rate (Fig. S5a†). However, at the low shear flow rate of 0.017 mL min^−1^, increasing the silk concentration from 0.5% to 2% and 3% reduced the nanoparticle α-helix and random coil content from 25% to 19% (Fig. S5a†). Conversely, in the unstirred condition, the nanoparticle correlation coefficients decreased as the concentration increased from 0.5% to 2% and 3%, at 0.017 mL min^−1^ (from 0.27 to 0.15 and 0.15), 1.000 mL min^−1^ (from 0.33 to 0.18 and 0.20) and 8.485 mL min^−1^ (from 0.31 to 0.21 and 0.15) (Fig. S5a†). This trend was supported by the significant increase in total β-sheet content with changes from 0.5 to 2% at 0.017 mL min^−1^ (58 to 60%) and from 0.5 to 2 and 3% at 8.48 mL min^−1^ (55 to 59 and 56%). Similarly, the intermolecular β-sheet content increased with silk feed concentration from 0.5 to 3% at 0.017 (34 to 42%). Additionally, for 3% silk feedstocks, the native β-sheet content significantly increased with flow rate whereas for 0.5% silk, the intermolecular β-sheet content significantly followed flow rate. Consequently, the nanoparticle α-helix and random coil content also decreased as the silk concentration increased from 0.5 to 3% at flow rates of 0.017 (20 to 19%), 1.000 (21 to 19%), and 8.485 mL min^−1^ (20 to 19%). Similarly, the extruded silk crystallinity increased with the feedstock concentration. For instance, increasing the concentration from 2 to 3% at 8.485 mL min^−1^ increased the extruded silk β-sheet content from 24% to 36% (Fig. S4e†). This result was paralleled by a reduction in the correlation coefficient as the concentration increased from 0.5%, at flow rates of 0.017 (0.90), 1.000 (0.85) and 16.96 mL min^−1^ (0.69), to 3.0%, at flow rates of 8.485 (0.32) and 16.96 mL min^−1^ (0.23) (Fig. S4e†).

The β-sheet content of 0.5% extruded silk increased with flow rate from 19% at 0.017 mL min^−1^ to 31% at 8.485 mL min^−1^ (Fig. S4e†). The interaction between flow rate and concentration also significantly increased the silk β-sheet content, from 19% to 29% and 37%, as the flow rate was increased from 0.017 mL min^−1^ to 16.96 mL min^−1^ and 16.96 mL min^−1^ at silk concentrations of 0.5%, 2.0% and 3.0%, respectively (Fig. S4e†). These results were supported by the reduction in the correlation coefficient of extruded silk from 0.78 to 0.32 and 0.23 as the flow rate increased from 0.017 mL min^−1^ to 8.485 mL min^−1^ and 16.96 mL min^−1^, respectively, at a silk concentration of 3.0% (Fig. S4e†).

In the micromixer, the nanoparticle β-sheet content did not significantly vary with silk concentration or flow rate, and the correlation coefficients displayed no general trend (Fig. S5b†). However, the nanoparticle α-helix and random coil content significantly increased with increases in silk concentration from 0.5 and 2 to 3% at 0.059 mL min^−1^ (from 20 and 19 to 28%) and decreased (from 22 to 19%) at 1.0 mL min^−1^ flow rate (Fig. S5b†). Further, the nanoparticle α-helix and random coil content generally increased with flow rate. For example, increasing the flow rate from 0.001 to 1.0 mL min^−1^ caused a significant increase from 18 to 22% for 0.5% silk feeds; by contrast, for 3% silk this content increased from 20 to 28% between 0.001 to 0.059 mL min^−1^ and decreased to 19% at 1.0 mL min^−1^.

## Discussion

In the current study, the optimised formulation variables^11–13^ for preparing silk nanoparticles were used to investigate the impact of flow and mixing on silk self-assembly. Consequently, all silk feed concentrations were below the ≈10% w/w critical micelle concentration^37^ of regenerated silk fibroin. We used the closed, semi-batch process for lab-scale nanoprecipitation, and the NanoAssemblr™ platform for the continuous process, with a commercially available staggered herringbone mixer Operating under conditions of laminar flow (*R*_e_ < 2000).

### The effect of flow rate, addition height and stirring rate in closed, semi-batch format

The flow rates between 0.017 and 16.96 mL min^−1^ resulted in Reynolds numbers <2000 within the tubing and with all needles used; hence, laminar and newtonian flow was used to calculate the average wall shear rate.^37^ The average wall shear rates in the syringe and tubing (Table 1), combined with the residence times, were not expected to provide sufficient work (≈10^5^ Pa)^14^ for shear-induced nucleation of the silk molecules at flow rates between 0.017 and 16.96 mL min^−1^. However, conditions of high shear were introduced at flow rates above 0.017 mL min^−1^ in needles with diameters less than 1.6 mm (Table 1).

**Fig. 6:**
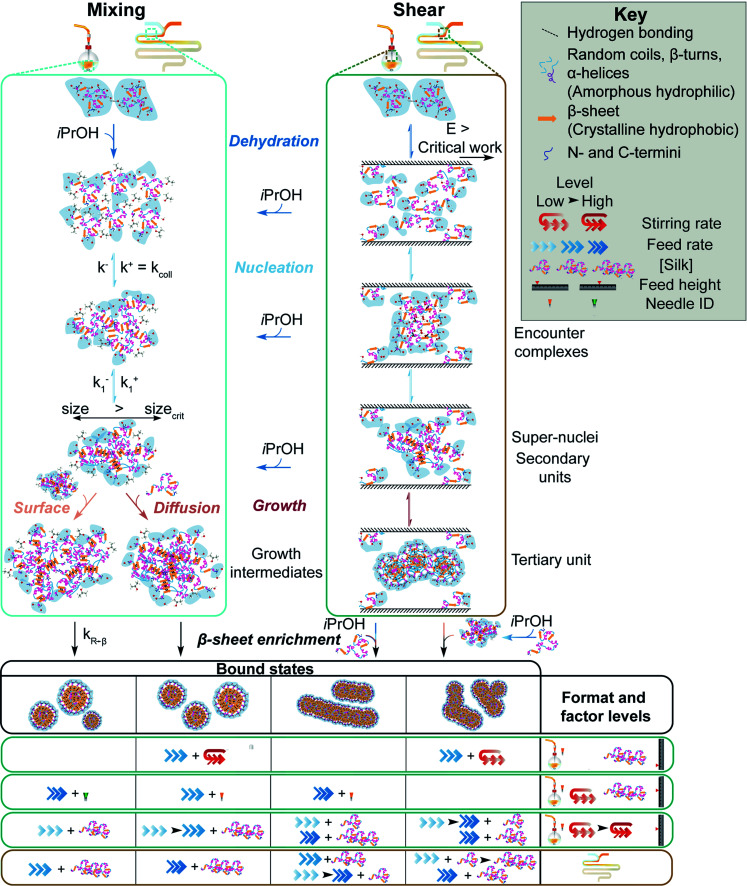
Schematic of protein–protein association and β-sheet assembly of silk fibroin *via* anti-solvent and shear-induced desolvation. The mechanism under shear flow is adapted from Dunderdale *et al.*^14^ and Zhang *et al.*^54^ Silk molecules, nanoparticles and stoichiometry of association are not drawn to scale.

Regenerated silk fibroin can undergo a primary assembly process into silk fibroin micellar structures (secondary building units), followed by secondary and tertiary assembly of the micellar units into nanofibers and lamellar structures (Fig. 6).^54^ The hydrophilic blocks of the silk fibroin polymer are hydrated in aqueous solution, resulting in an extended conformation.^54^ The shear-induced desolvation breaks the stabilizing intermolecular bonds with water and enables hydrophobic interactions between silk molecules.^54^ Scanning electron microscopy showed that shear-induced nucleation resulted in secondary and tertiary assembly of silk micelles into nanofibres and lamellar structures, prior to antisolvent addition and completion of mixing (Fig. S2c, d† and 3b).^55^ This was reinforced by the significantly lower correlation coefficients of the silk extruded from the 0.33 mm needle at all flow rates above 0.017 mL min^−1^ (Fig. S4b†).

At all feed heights, mixing of silk within free falling droplets was driven by convection due to the high fluid velocities (Table S4†). Consequently, the impact of non-uniform silk concentration on solvent-antisolvent mixing efficiency can be neglected. However, for flow conditions of high shear, self-assembly within the droplet during flight could occur due to the microscale diffusion length of the silk molecules (Table S4†). The incorporation of water into, and size of, shear-induced silk assemblies would increase with feed height and time of flight. The concomitant reduction in free water concentration within the droplet could have reduced the solvent-antisolvent mixing efficiency and resulted in large, polydisperse assemblies (Fig. S2†).

Without stirring, at a 1.000 mL min^−1^ feed rate and 0.0 cm height, the shear-induced nucleation, and the antisolvent-induced dehydration resulted in nanoparticle formation at the phase boundary upon addition of the aqueous silk feed to the isopropanol bulk. This resulted in phase separation and low reproducibility due to the uncontrolled manual mixing of the two phases during purification. Increasing the stirring rate and feed height increased the rate and degree of antisolvent-induced nucleation by reducing the mixing time (Table 2).

Assembly growth was disfavoured under conditions of low mixing time, resulting in low nanoparticle size and polydispersity with increased yield. Additionally, the shear stress of the liquid flow arising from stirring rates and feed heights of 200 rpm and 1.75 cm could exceed the shear stress of agglomerates formed during silk extrusion from the feed needle. Breaking the intermolecular bridges holding the agglomerates together, combined with desolvation, would result in kinetic locking of the secondary building units. This was supported by the increase of nanoparticle β-sheet content with feed height (Fig. S4d†) and the low curvature morphology of tertiary assemblies formed at low heights and stirring rates (Fig. 3b).

### The effect of flow rate and concentration in closed semi-batch and microfluidic formats

In the stirred semi-batch process, at feed rates of 0.017, 1.000, 8.485 and 16.96 mL min^−1^, the shear rates experienced by silk in the fluid line were estimated as 0.18, 10, 88 and 176 s^−1^, respectively, while the shear rates in the needle were estimated as 80, 4724, 40 083 and 80 114 s^−1^ (Tables S2† and 1). At 1.000 mL min^−1^ and above, the needle shear rates lay above the critical shear rate and work required for nucleation.^56^ Shear-induced nucleation was reinforced by a decrease in the correlation coefficients of extruded silk as the flow rate increased across all concentrations and an increase in the β-sheet content of extruded silk as the flow rate increased at 0.5 and 2.0% (Fig. S4e†). Consequently, the following arguments assume that shear-induced nucleation, followed by isopropanol-induced desolvation, occurred above a flow rate of 0.017 mL min^−1^.

For homogenous nucleation at 0.017 mL min^−1^, increasing the concentration from 0.5 to 3.0% reduced the assembly size and polydispersity index due to a greater degree of supersaturation in both low and high mixing time processes (Fig. 4a). Samples prepared using 3.0% silk at flow rates of 0.017 and 1.000 mL min^−1^ and stirring at 400 rpm could be considered monodisperse. At 0.017 mL min^−1^, the reduction in the growth phase with increasing concentration significantly increased the assembly curvature (Fig. 5a) and packing due to decreases in the α-helix and random coil contents (Fig. S5a†). The reduced growth observed at higher silk concentrations in low-shear, semi-batch nanoprecipitation contrasts with results of previous work by D. Pham *et al.*,^31^ who used lower antisolvent:silk volumetric ratios.

In the unstirred system, under high-shear conditions and at all flow rates exceeding 0.017 mL min^−1^, increasing the silk feed concentration from 0.5 to 3.0% significantly reduced the particle size, whereas in the stirred system, the decreases in size were not significant (Fig. 4a). A mixture of low sphericity particles and aggregates were formed under high shear at 0.5 and 2.0%, while a mixture of nanofibres, lamellar structures and spherical particles were formed at 3.0% (Fig. 5a). This possibly reflected a balance between increased antisolvent-induced nucleation rate and increased rates of shear-induced self-assembly with concentration. The greater degree of shear-induced self-assembly with increasing concentration, from 0.5 to 3.0%, was reinforced by the increase in total and intermolecular β-sheet contents of the nanoparticles, the increased total β-sheet content of the extruded silk, the reduction in the nanoparticle correlation coefficients with concentration and the decrease in extruded silk correlation coefficients at 8.485 and 16.96 mL min^−1^ (Fig. S4e†). Consequently, as the formation of shear-induced micelles increased with feed rate, the reduced silk concentration prohibited antisolvent-induced nucleation.

The feed rate of silk also impacts mixing-induced self-assembly by altering the mixing time. For example, the droplet surface area decreased as the feed rate increased from 0.017 to 1.000 mL min^−1^ and then increased as the feed rate was raised to 16.96 mL min^−1^ (Table S4†). The reduced sphericity of the droplets produced in the jetting regime above 7.000 mL min^−1^ (Fig. 2a) could also cause non-uniform mixing. The effect of decreasing the mixing time as the feed rate was increased could be observed in the unstirred system at 0.5% and 2% silk feed concentrations. At these concentrations, the nanoparticle size and polydispersity index decreased with flow rate because the increase in the antisolvent-induced nucleation rates outweighed the increase in shear-induced assembly rates. Further, the feed rate controls the degree and rate of supersaturation onset of the antisolvent-induced desolvation. This means that the type and rate of antisolvent-induced nucleus growth could be impacted by the rate of silk addition. Increasing the feed rate favours mononuclear surface-controlled growth by increasing the mass transfer of silk from the solution to the assembly surface. For example, as the feed rate increased in the stirred semi-batch process for 2% silk feeds, the resulting assemblies increased in size, size distribution and zeta potential (Fig. 4a).

Continuous manufacture is a well-recognised strategy that simplifies the scale-up process of nanoformulations.^57^ We therefore used the NanoAssemblr™ as an example of a scale-independent process. Assuming newtonian flow, the flow rates of 0.001 mL min^−1^ to 1 mL min^−1^ resulted in Reynolds numbers of 0.04 to 40. These numbers were below the high vorticity and low transverse flow regime (Reynolds numbers > 1000). This ensured a high mixing efficiency, with the mixing time decreasing as flow rate increased from 0.001 to 1.0 mL min^−1^. The residence times at all flow rates were longer than the mixing times (Table 2), indicating that complete mixing occurred in the micromixer. The wall shear rates increased with flow rate, and when combined with the residence time, shear-induced nucleation of silk fibroin was likely to occur at all flow rates greater than 0.001 mL min^−1^, as both the critical shear rate^56^ and work^14^ were exceeded. Because the shear rates in both formats were analogous, the effects of shear and mixing in the micromixer could be compared with semi-batch processes with low and high mixing times (Fig. 6).

In the micromixer and with 0.5% silk feeds, as the shear rate and mixing efficiency increased with flow rate from 0.001 to 1.0 mL min^−1^, the assembly morphology shifted from amorphous aggregates to mixtures of rod-like and spherical particles. We speculate that the observation of rod-like morphologies only with 0.5% silk feeds was due to the low degrees of nucleation and long shear-induced and antisolvent-induced growth phases associated with insufficient surpassing of the nucleation energy barrier (Fig. 5b). The size reduction in the nanoassemblies with increasing flow rate indicated that the enhancement of the antisolvent-induced nucleation rates dominated over the increase in shear-induced assembly rates.

The association and assembly processes were favoured by an increase in the silk concentration from 0.5 to 2%,^54^ which resulted in the formation of larger nanofibres and lamellar tertiary structures with a higher polydispersity index under homogenous nucleation at 0.001 mL min^−1^ (Fig. 4b and 5b). This effect indicated that the increase in the silk concentration still did not greatly exceed the nucleation barrier. The opposite trend was observed for semi-batch processes at low and high mixing times due to the higher saturation induced by larger initial antisolvent-to-silk ratios.

Following the increase in flow rate from 0.001 to 0.059 mL min^−1^ for 2% feeds, the reduction in the mixing time dominated over the increase in shear-induced nucleation and assembly (Fig. 4b). The resulting particles showed spherical morphologies, although shear-induced assembly also resulted in the secondary assembly of nanoparticles into nanofibres. However, for 2% silk, further increases in the flow rate produced nanoparticles of increasing size due to secondary and tertiary growth processes that gave rise to amorphous and fibre-like assemblies. This trend was also observed for the low-mixing-time, semi-batch system, supporting the idea that the increase in the rates of shear-induced nucleation and growth in the microchannel dominated over the effect of reduced mixing time.

A silk concentration increase from 2 to 3%, under homogeneous nucleation at 0.001 mL min^−1^, significantly reduced both the nanoparticle size and the polydispersity index due to an increase in supersaturation and the antisolvent-induced nucleation rates. Increasing the silk concentration to 3% caused gradual increases in viscosity and rapid decreases in the surface tension.^56,58^ We speculate that these changes increased the water transport rate between the aqueous and isopropanol phases for 3% silk feeds, thereby providing less time for secondary self-assembly processes.

The extent of self-assembly for 3% silk feedstocks decreased as the flow rate increased from 0.001 to 1 mL min^−1^, with reductions in nanoparticle size, polydispersity index and zeta potential, but particles were produced in greater yield. The lower degree of self-assembly with increasing flow rate was confirmed by the increase in particle curvature (Fig. 5b) and the increase in α-helix and random coil content (Fig. S5b†). Increasing the silk feed concentration from 0.5 and 2% to 3% reduced the critical shear rate.^56^ Consequently, at flow rates above 0.001 mL min^−1^ in the micromixer, the shear-induced nucleation rates for 3% silk were likely greater than those for 0.5 and 2% silk feeds. As the flow rate increased, the assembly growth phase period for 3% silk was shortened due to the higher shear-induced nucleation rates, combined with the increased rates of antisolvent-induced nucleation and kinetic locking of the micellar structure. The narrower growth phases compared to the lower silk concentrations were supported by the greater nanoparticle sphericities for 3% feeds. This trend was not observed with 3% silk for either the low-mixing-time or high-mixing-time semi-batch processes.

The dependence of particle morphology and properties on the flow rate through the microchannel was supported by previous demonstrations in the staggered herringbone micromixer.^12^ The kinetic freezing of secondary building units observed with 3% silk at the 1 mL min^−1^ flow rate agreed with previous literature reports.^12,13^ Further, for 3% silk feeds, secondary and tertiary self-assembly were promoted by shear processing following an increase in the flow rate from 1 to 12 mL min^−1^ (shear rate 961 499 s^−1^), resulting in reduced particle curvature and increased overall size and polydispersity.^12^ Consequently, a critical flow rate exists between 1 and 12 mL min^−1^ for 3% silk feeds, beyond which the increase in the antisolvent-induced nucleation rate is out-competed by the increase in the rate of shear-induced assembly. This non-linear trend of self-assembly reflects the trend observed for 2% silk, but was shifted to higher flow rates due to the shortened growth phase occurring from greater supersaturation.

## Conclusions

The flow properties of silk fibroin, which are fundamental to the natural role of the polymer as a polymorphic material, can be exploited in nanoprecipitation processes. Here, to control the morphology of silk nanoparticles, we utilised shear-induced nucleation of silk fibroin under flow in semi-batch and microfluidic formats. The morphology of the resulting silk assemblies varied with shear processing and silk precursor concentration in bulk mixing, although nanofibre and lamellar assemblies were formed as mixtures with spherical particles. Conversely, the high-shear, low-mixing-time conditions provided by the micromixer identifies this as a promising platform for tuning primary–tertiary silk self-assembly. Due to the sufficiently low mixing time, the silk concentration could be used as a controllable input factor for the formation of monodisperse, rod-like and spherical nanoassemblies suitable for use as nanomedicines. The information provided in this study delineates rational guidelines for the modulation of silk fibroin multiscale structures under shear and antisolvent-induced desolvation by varying the supersaturation, shear rate and mixing time.

## Author contributions

S. A. L. M. designed, collected, analysed, and interpreted the data and generated the manuscript draft. R. R. conducted silk concentration experiments at 1 mL min^−1^ using microfluidics and interpreted results. J. K. collected SEM images and interpreted results. Y. P. and F. P. S. provided training, advised on experimental design, and contributed to the interpretation of the results. All authors discussed the results and/or provided advice on the experimental analysis. F. P. S. supervised the project and content-edited the manuscript.

## Conflicts of interest

There are no conflicts to declare.

## Supplementary Material

RA-012-D1RA07764C-s001

## References

[cit1] Konwarh R. (2019). Bio-Des. Manuf..

[cit2] Chouhan D., Mandal B. B. (2020). Acta Biomater..

[cit3] Maitz M. F., Sperling C., Wongpinyochit T., Herklotz M., Werner C., Seib F. P. (2017). Nanomedicine.

[cit4] Lozano-Pérez A. A., Montalbán M. G., Aznar-Cervantes S. D., Cragnolini F., Cenis J. L., Víllora G. (2015). J. Appl. Polym. Sci..

[cit5] Holland C., Numata K., Rnjak-Kovacina J., Seib F. P. (2019). Adv. Healthcare Mater..

[cit6] Xiao L., Lu G., Lu Q., Kaplan D. L. (2016). ACS Biomater. Sci. Eng..

[cit7] Mehrotra S., Chouhan D., Konwarh R., Kumar M., Jadi P. K., Mandal B. B. (2019). ACS Biomater. Sci. Eng..

[cit8] Wenk E., Merkle H. P., Meinel L. (2011). J. Controlled Release.

[cit9] Tomeh M. A., Hadianamrei R., Zhao X. (2019). Pharmaceutics.

[cit10] Jin H.-J., Kaplan D. L. (2003). Nature.

[cit11] Wongpinyochit T., Johnston B. F., Seib F. P. (2016). J. Visualized Exp..

[cit12] Wongpinyochit T., Totten J. D., Johnston B. F., Seib F. P. (2019). Nanoscale Adv..

[cit13] Solomun J. I., Totten J. D., Wongpinyochit T., Florence A. J., Seib F. P. (2020). ACS Biomater. Sci. Eng..

[cit14] Dunderdale G. J., Davidson S. J., Ryan A. J., Mykhaylyk O. O. (2020). Nat. Commun..

[cit15] Wacker M. (2013). Int. J. Pharm..

[cit16] Richtering W., Alberg I., Zentel R. (2020). Small.

[cit17] Arno M. C., Inam M., Weems A. C., Li Z., Binch A. L. A., Platt C. I., Richardson S. M., Hoyland J. A., Dove A. P., O'Reilly R. K. (2020). Nat. Commun..

[cit18] Zhao J., Stenzel M. H. (2018). Polym. Chem..

[cit19] Crivelli B., Perteghella S., Bari E., Sorrenti M., Tripodo G., Chlapanidas T., Torre M. L. (2018). Soft Matter.

[cit20] Gupta V., Aseh A., Ríos C. N., Aggarwal B. B., Mathur A. B. (2009). Int. J. Nanomed..

[cit21] Seib F. P., Jones G. T., Rnjak-Kovacina J., Lin Y., Kaplan D. L. (2013). Adv. Healthcare Mater..

[cit22] Wongpinyochit T., Uhlmann P., Urquhart A. J., Seib F. P. (2015). Biomacromolecules.

[cit23] Zhao Z., Li Y., Xie M.-B. (2015). Int. J. Mol. Sci..

[cit24] Gholami A., Tavanai H., Moradi A. R. (2011). J. Nanopart. Res..

[cit25] Toprakcioglu Z., Challa P. K., Morse D. B., Knowles T. (2020). Sci. Adv..

[cit26] Kazemimostaghim M., Rajkhowa R., Wang X. (2015). Powder Technol..

[cit27] Lu Q., Huang Y., Li M., Zuo B., Lu S., Wang J., Zhu H., Kaplan D. L. (2011). Acta Biomater..

[cit28] Tarhini M., Greige-Gerges H., Elaissari A. (2017). Int. J. Pharm..

[cit29] Lepeltier E., Bourgaux C., Couvreur P. (2014). Adv. Drug Delivery Rev..

[cit30] Botet R., Roger K. (2016). Curr. Opin. Colloid Interface Sci..

[cit31] Pham D. T., Saelim N., Tiyaboonchai W. (2018). Int. J. Appl. Pharm..

[cit32] Breslauer D. N., Muller S. J., Lee L. P. (2010). Biomacromolecules.

[cit33] Shimanovich U., Ruggeri F. S., De Genst E., Adamcik J., Barros T. P., Porter D., Müller T., Mezzenga R., Dobson C. M., Vollrath F., Holland C., Knowles T. P. J. (2017). Nat. Commun..

[cit34] Toprakcioglu Z., Levin A., Knowles T. P. J. (2017). Biomacromolecules.

[cit35] Kee S. P., Gavriilidis A. (2008). Chem. Eng. J..

[cit36] Matthew S. A. L., Totten J. D., Phuagkhaopong S., Egan G., Witte K., Perrie Y., Seib F. P. (2020). ACS Biomater. Sci. Eng..

[cit37] Nisal A., Kalelkar C., Bellare J., Lele A. (2013). Rheol. Acta.

[cit38] Ianovska M. A., Mulder P. P. M. F. A., Verpoorte E. (2017). RSC Adv..

[cit39] Rode García T., García Ac A., Lalloz A., Lacasse F.-X., Hildgen P., Rabanel J.-M., Banquy X. (2018). Langmuir.

[cit40] Melton L. A., Lipp C. W., Spradling R. W., Paulson K. A. (2002). Chem. Eng. Commun..

[cit41] Weheliye W. , Rodriguez G., Anderlei T., Micheletti M., Yianneskis M. and Ducci A., in Proceedings of the 14th European Conference on Mixing, Warszawa, Warsaw, Poland, 2012, pp. 503–508

[cit42] Rodriguez G., Weheliye W., Anderlei T., Micheletti M., Yianneskis M., Ducci A. (2013). Chem. Eng. Res. Des..

[cit43] Tomar S. (2006). Linux J..

[cit44] Rodriguez G., Anderlei T., Micheletti M., Yianneskis M., Ducci A. (2014). Biochem. Eng. J..

[cit45] Jiang X., Zheng L., Wu H., Zhang J. (2021). Math. Biosci. Eng..

[cit46] Xu Z., Lu C., Riordon J., Sinton D., Moffitt M. G. (2016). Langmuir.

[cit47] Pang F.-M., Seng C.-E., Teng T.-T., Ibrahim M. H. (2007). J. Mol. Liq..

[cit48] Williams M. S., Longmuir K. J., Yager P. (2008). Lab Chip.

[cit49] Mialdun A., Yasnou V., Shevtsova V., Königer A., Köhler W., Alonso De Mezquia D., Bou-Ali M. M. (2012). J. Chem. Phys..

[cit50] Son Y. (2007). Polymer.

[cit51] Yang H., Yang S., Kong J., Dong A., Yu S. (2015). Nat. Protoc..

[cit52] Hu X., Kaplan D., Cebe P. (2006). Macromolecules.

[cit53] Griebenow K., Santos A. M., Carrasquillo K. G. (1999). Internet J. Vib. Spectrosc..

[cit54] Zhang Y., Zuo Y., Wen S., Hu Y., Min Y. (2018). Biomacromolecules.

[cit55] Zainuddin, Le T. T., Park Y., Chirila T. V., Halley P. J., Whittaker A. K. (2008). Biomaterials.

[cit56] Matsumoto A., Lindsay A., Abedian B., Kaplan D. L. (2008). Macromol. Biosci..

[cit57] Webb C., Forbes N., Roces C. B., Anderluzzi G., Lou G., Abraham S., Ingalls L., Marshall K., Leaver T. J., Watts J. A., Aylott J. W., Perrie Y. (2020). Int. J. Pharm..

[cit58] Chung D. E., Um I. C. (2014). Fibers Polym..

