# Correction: Mixing and flow-induced nanoprecipitation for morphology control of silk fibroin self-assembly

**DOI:** 10.1039/d2ra90071h

**Published:** 2022-09-07

**Authors:** Saphia A. L. Matthew, Refaya Rezwan, Jirada Kaewchuchuen, Yvonne Perrie, F. Philipp Seib

**Affiliations:** Strathclyde Institute of Pharmacy and Biomedical Sciences, University of Strathclyde 161 Cathedral Street Glasgow G4 0RE UK philipp.seib@strath.ac.uk +44 (0)141 548 2510; Department of Pharmacy, ASA University Bangladesh 23/3 Bir Uttam A. N. M. Nuruzzaman Sarak Dhaka 1207 Bangladesh; EPSRC Future Manufacturing Research Hub for Continuous Manufacturing and Advanced Crystallisation (CMAC), University of Strathclyde, Technology and Innovation Centre 99 George Street Glasgow G1 1RD UK; Faculty of Nursing, HRH Princess Chulabhorn College of Medical Science, Chulabhorn Royal Academy Bangkok Thailand

## Abstract

Correction for ‘Mixing and flow-induced nanoprecipitation for morphology control of silk fibroin self-assembly’ by Saphia A. L. Matthew *et al.*, *RSC Adv.*, 2022, **12**, 7357–7373. https://doi.org/10.2039/D1RA07764C.

The authors regret that there were sub-figure placement errors present in Fig. 4 and 5 of the main article. The sub-figure placement error in Fig. 4 was carried into Fig. S3, which shows additional statistical significances. The corrected figures are shown below.

Consequently, sections of the text in the manuscript should be adjusted according to this change, and these are detailed below:

On page 7365, the sentence starting: “Under conditions of mixing induced nanoprecipitation in semi-batch format…”, should be given as “Under conditions of mixing-induced nanoprecipitation in semi-batch format at a 0.017 mL min^−1^ flow rate and 400 rpm stirring rate, increasing the silk concentration from 0.5 to 2 and 3% significantly decreased the nanoparticle size (from 271 to 89 and 75 nm) and polydispersity index (from 0.47 to 0.23 and 0.13) (Fig. 4a and S3a). Significant decreases in the nanoparticle size (from 294 to 161 and 120 nm), polydispersity index (from 0.46 to 0.41 and 0.24) and zeta potential magnitude (from −39 and −40 to −30 mV) also occurred with a concentration increase from 0.5 to 2 and 3% when operating without stirring.”

On page 7365 the sentence starting: “At the 400 rpm stirring rate…”, should be given as “At the 400 rpm stirring rate, moving from low to high shear by increasing the flow rate from 0.017 to 16.96 mL min^−1^ caused a significant increase in the nanoparticle size from 89 to 252 nm with 2% silk. The size distribution also significantly increased with flow rate at silk concentrations of 0.5 and 2%.”

On page 730 the sentence starting: “For example, as the feed rate increased…” should be given as “For example, as the feed rate increased in the stirred semi-batch process for 2% silk feeds, the resulting assemblies increased in size and size distribution (Fig. 4a).”

Accordingly, Fig. S3 in the original ESI should be replaced with the following revised Fig. S3. The ESI has been updated online to reflect this change.

Fig. 4


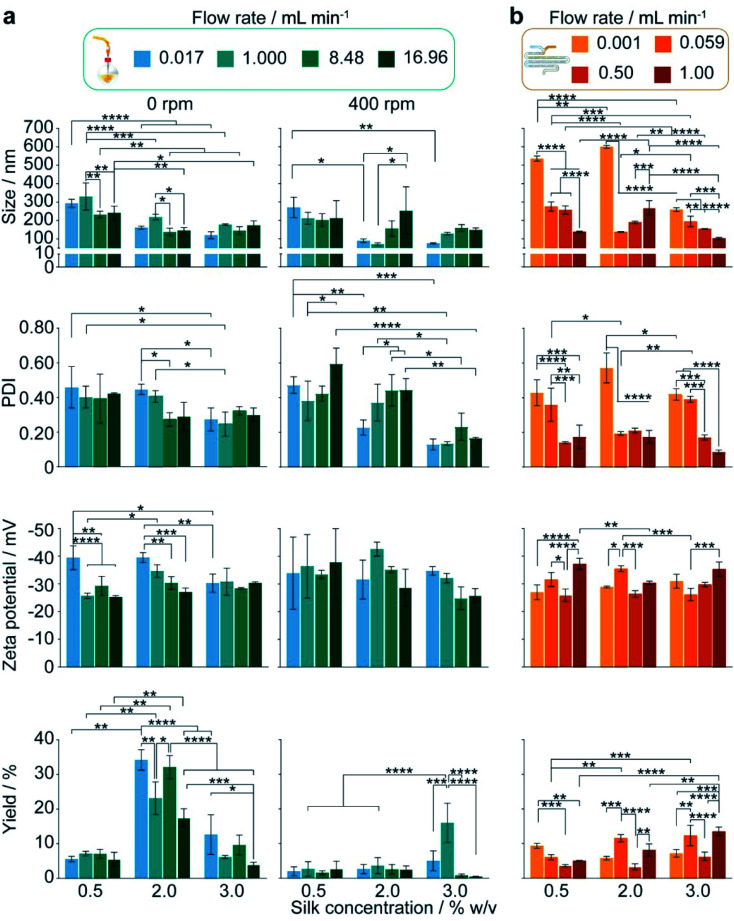


Fig. 5


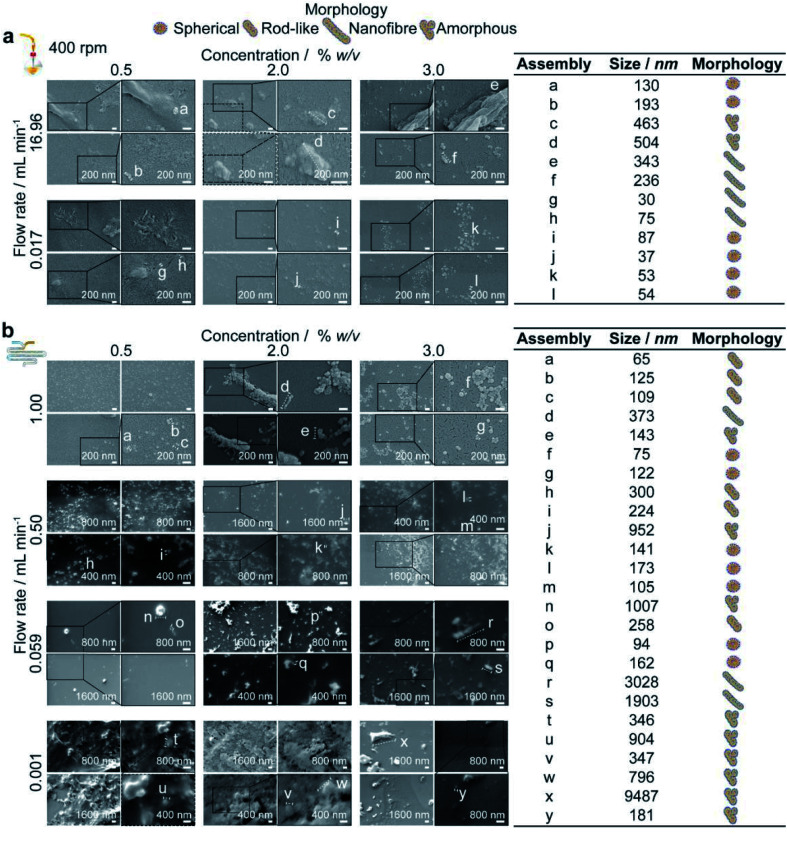


Fig. S3


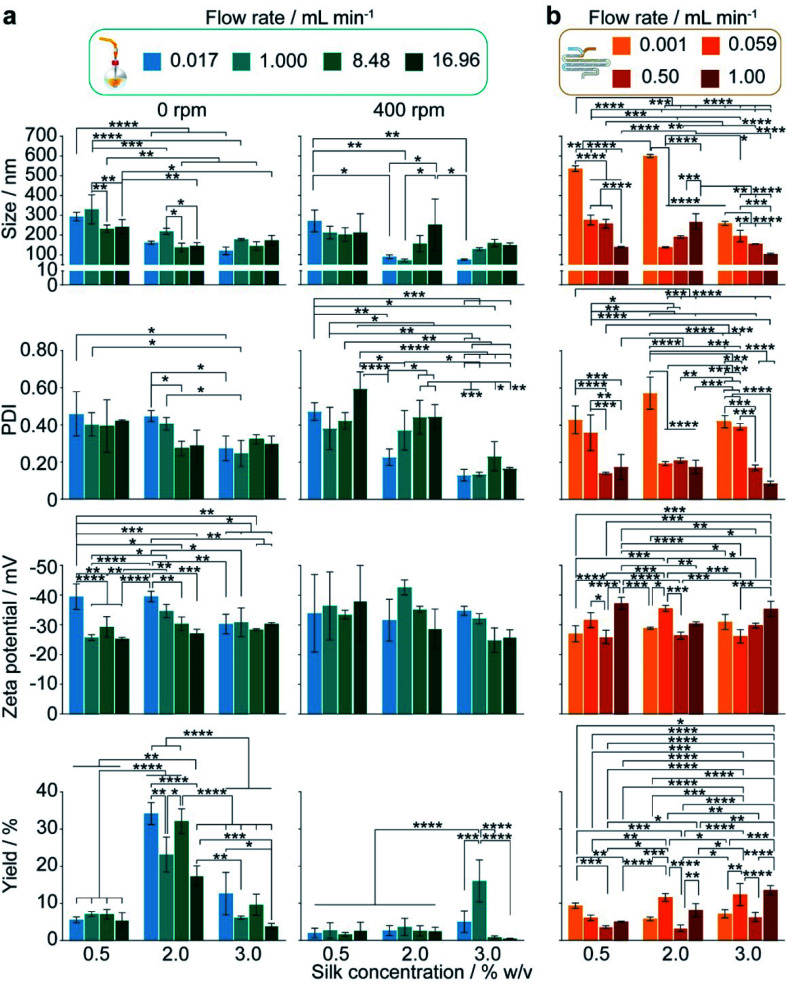


## Supplementary Material

