# Supplementary material for: Mixing and flow-induced nanoprecipitation for morphology control of silk fibroin self-assembly
Source: RSC Adv. 2022 Mar 4;12(12):7357–73. doi: 10.1039/d1ra07764c (PMC8982335; doi:10.1039/d1ra07764c)
Supplement: RA-012-D1RA07764C-s001 [file RA-012-D1RA07764C-s001.pdf]

Electronic Supplementary Material (ESI) for RSC Advances.
This journal is © The Royal Society of Chemistry 2022

Supporting Information

Saphia A. L. Matthew,[†] Refaya Rezwan[‡], Jirada Kaewchuchuen,[†, §] Yvonne Perrie[†] and F. Philipp Seib[, †, $]*

* Corresponding author: Tel: +44 (0) 141 548 2510; E-mail: philipp.seib@strath.ac.uk.

† Strathclyde Institute of Pharmacy and Biomedical Sciences, University of Strathclyde, 161 Cathedral Street, Glasgow, G4 0RE, U.K.

‡ Department of Pharmacy, ASA University Bangladesh, 23/3 Bir Uttam A.N.M Nuruzzaman Sarak, Dhaka 1207, Bangladesh.

§ Faculty of Nursing, HRH Princess Chulabhorn College of Medical Science, Chulabhorn Royal Academy, Bangkok, Thailand.

$ EPSRC Future Manufacturing Research Hub for Continuous Manufacturing and Advanced Crystallisation (CMAC), University of Strathclyde, Technology and Innovation Centre, 99 George Street, Glasgow G1 1RD, U.K.

Number of pages: S1-S9

Number of figures: S1-S5

Number of tables: S1-S4

Table S1. Estimated flow characteristics of the syringes used in the closed semi-batch system indicated that shear-induced nucleation did not occur.

Syringe / *mL*	Volume / *mL*	Flow rate / *mL min⁻¹*	Residence time / *s*	Critical work / *10⁻³ Pa*	Maximum shear rate / *s⁻¹*	Volumetric flow rate / *10⁻¹⁰ m³ s*	Average velocity / *10⁻⁵ m s⁻¹*	Cross-sectional area / *10⁻⁵ m²*	R_e
3	1	0.017	3529	1.88	0.00444	2.83	0.481	5.89	0.00157
		1.000	18	33.2	0.261	167	28.3		0.093
		3.510	17	389	0.917	585	99.3		0.325
		7.000	9	775	1.83	1167	198		0.648
		8.485	7.07	1.09	0.0757	1414	25.3	56.0	0.255
		16.96	3.54	2.19	0.151	2827	50.5		0.509

Table S2. Estimated flow characteristics of the fluid line used in the closed semi-batch system indicated that shear-induced nucleation did not occur.

**Flow rate / *mL min⁻¹***	**Residence time / *ms***	**Critical work / *10³ Pa***	**Maximum shear rate / *s⁻¹***	**Volumetric flow rate / *10⁻¹⁰ m³ s***	**Average velocity / *10⁻⁵ m s⁻¹***	**Cross-sectional area / *10⁻⁶ m²***	**R_e**
0.017	5451	0.0046	0.18	2.83	5.59	5.07	0.0054
1.000	93	0.27	10	167	329		0.316
3.510	26	0.94	36	585	1155		1.108
7.000	13	1.90	73	1167	2302		2.209
8.485	11	2.3	88	1414	2791		2.678
16.96	5	4.6	176	2827	5578		5.353

Table S3. Estimated flow characteristics of the reactors used in the closed semi-batch system.

**Stirring rate / *rpm***	**Frequency / *rps***	**Length / *m***	**Diameter / *m***	**Viscosity / *kg ms⁻¹***	**Density / *kg m⁻³***	**R_e**
200	21	0.015	0.0060	0.0032	837	1257
400	42					2514
800	84					5029

Table S4. Droplet characteristics impacting flow and mixing-induced silk self-assembly indicated that circulatory flow occurred across all factor combinations, resulting in convection dominating over diffusion. ± SD, *n = 3.*

**Height / *cm***	**Flow rate / *mL min⁻¹***	**Number of drops / *mL⁻¹***	**Droplet Volume / *µL***	**Droplet diameter / *mm***	**Diffusion length scale[a] / *µm***	**Diffusion time scale[a] / *s***	**Time of flight / *s***	**Droplet velocity / *m s⁻¹***	**Fluid velocity / *mm s⁻¹***
0	0.017	NA		0.33	NA	7.42	NA	0.020 ± 0.007	8 ± 5
	1.000							0.028 ± 0.010	16 ± 12
	8.485							0.066 ± 0.072	38 ± 27
	16.96							0.056 ± 0.022	36 ± 23
0.7	3.510	28 ± 2	36.3 ± 3.2	2.73 ± 0.16	18.9 ± 4.1	508 ± 59.2	0.025 ± 0.011	0.335 ± 0.007	26 ± 19
	7.000	3 ± 1	305.6 ± 48.1	2.76 ± 0.17	14.2 ± 1.2	521 ± 63.9	0.014 ± 0.002	0.203 ± 0.016	35 ± 16
1.75	0.017	126 ± 2	7.9 ± 0.1	2.43 ± 0.04	31.3 ± 0.0	404 ± 13.1	0.067 ± 0	0.373 ± 0.062	12 ± 6
	1.000	33 ± 4	31.0 ± 4.1	2.59 ± 0.12	27.8 ± 0.6	457 ± 41.2	0.053 ± 0.002	0.344 ± 0.130	13 ± 12
	8.485	98 ± 17	10.5 ± 2.0	3.96 ± 0.63	24.8 ± 4.9	1090 ± 354	0.047 ± 0.016	0.392 ± 0.032	29 ± 13
	16.96	92 ± 55	15.1 ± 11.2	3.38 ± 0.97	25.8 ± 3.2	833 ± 446	0.046 ± 0.011	0.240 ± 0.146	33 ± 19
2.1	3.510	28 ± 2	36.3 ± 3.2	3.44 ± 0.15	27.4 ± 1.3	809 ± 69.9	0.051 ± 0.004	0.490 ± 0.005	29 ± 17
	7.000	148 ± 20	6.8 ± 0.9	2.51 ± 0.78	26.3 ± 1.8	464 ± 271	0.053 ± 0.006	0.425 ± 0.006	28 ± 13
3.5	0.017	126 ± 2	7.9 ± 0.1	2.24 ± 0.05	40.3 ± 3.4	343 ± 14.1	0.111 ± 0.019	0.530 ± 0.005	22 ± 12
	1.000	33 ± 4	31.0 ± 4.1	2.71 ± 0.39	32.9 ± 0.5	509 ± 147	0.074 ± 0.002	0.683 ± 0.003	29 ± 20
	3.510	28 ± 2	36.3 ± 3.2	3.42 ± 0.05	32.9 ± 0.5	799 ± 21.3	0.074 ± 0.002	0.642 ± 0.140	23 ± 6
	7.000	148 ± 20	6.8 ± 0.9	2.18 ± 0.79	31.6 ± 1.5	352 ± 266	0.074 ± 0.006	0.685 ± 0.019	50 ± 23
	8.485	88 ± 19	11.7 ± 2.3	3.82 ± 0.38	32.2 ± 2.6	1000 ± 195	0.068 ± 0.011	0.665 ± 0.033	37 ± 18
	16.96	149 ± 34	7.0 ± 1.8	1.97 ± 1.41	32.8 ± 2.2	377 ± 419	0.074 ± 0.010	0.731 ± 0.075	40 ± 18

a. Determined using the droplet diameter, droplet time of flight and the silk diffusion coefficient of 2.45 × 10^5 cm^2 s^{-1}.[1]

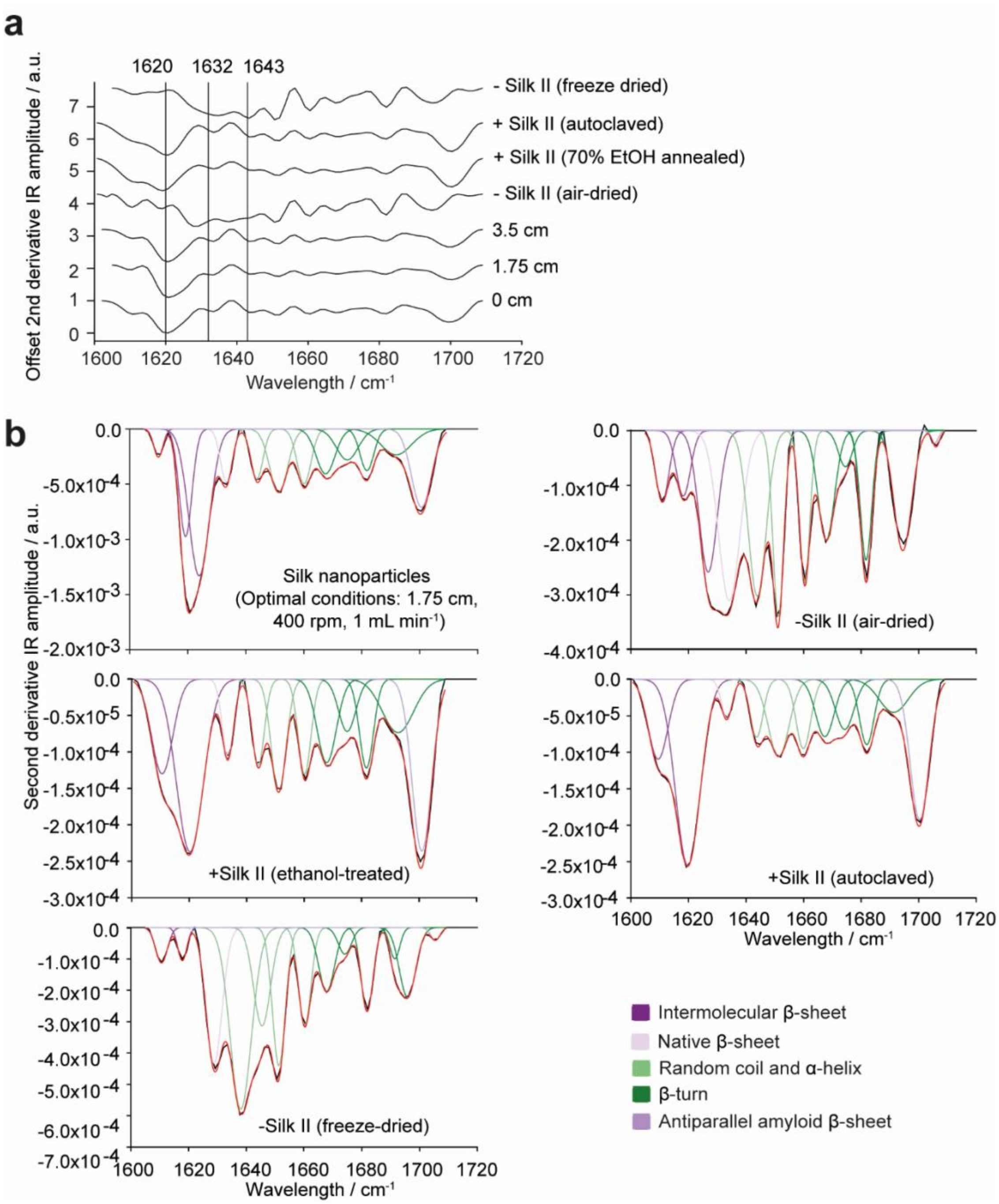

Figure S1. Exemplary smoothed second derivative FTIR spectra and peak assignment of silk nanoparticles manufactured by varying (a) feed addition height at a constant stirring rate of 400 rpm and a constant feed addition rate of 1 mL min^{-1} and (b) exemplary peak fitting of the amide I region for nanoparticles formulated at 1.75 cm feed height and silk II controls.

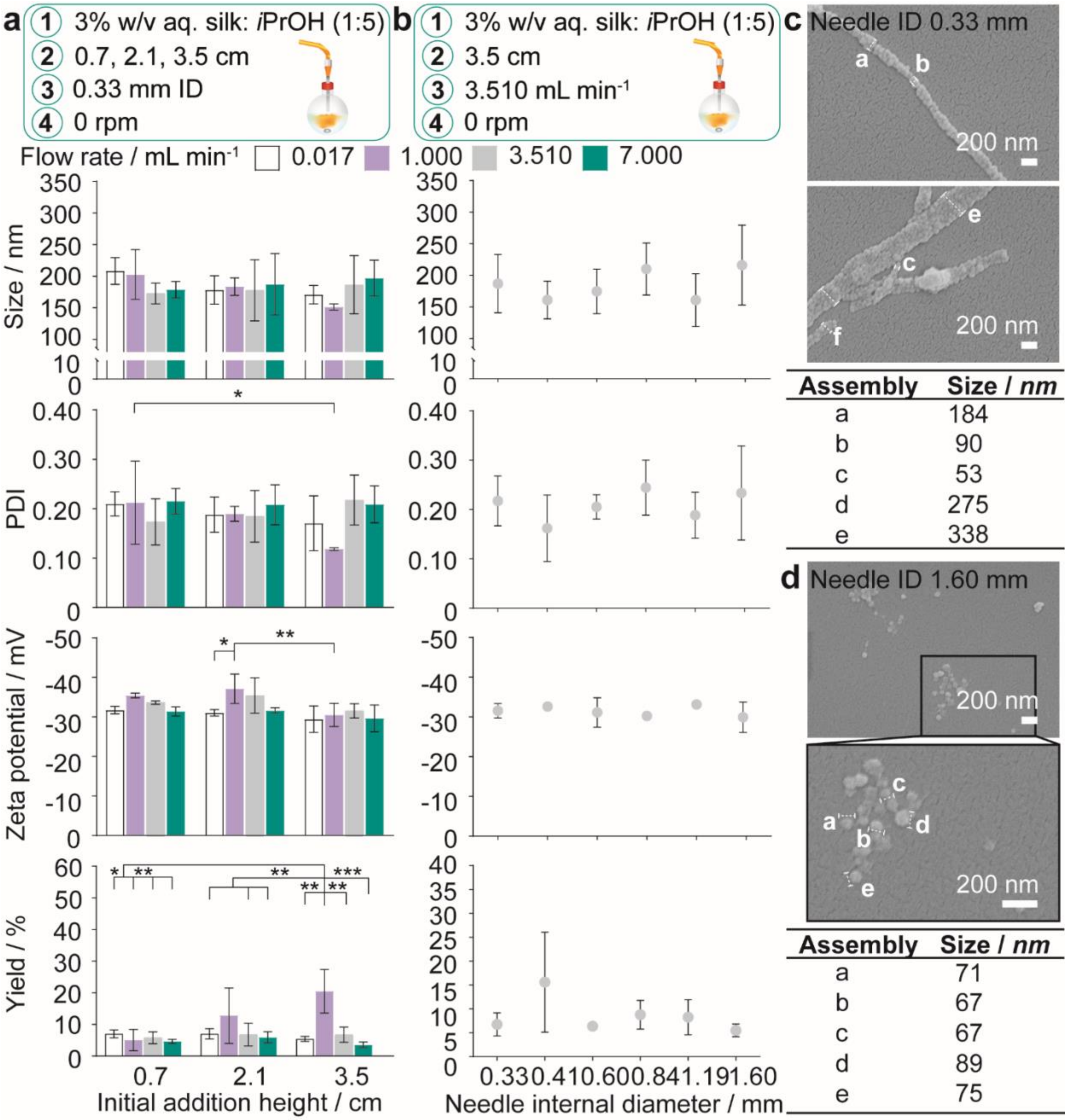

Figure S2. The impact in the drop-by-drop semi-batch closed-system by (a) initial addition height and flow rate using a constant needle diameter and by (c) needle diameter at constant flow rate and initial addition height on nanoparticle hydrodynamic diameter, polydispersity (PDI), zeta potential and yield. Scanning electron microscopy showed that curvature morphology increased with needle diameter from (c) 0.33mm to (d) 1.60 mm. Two-way ANOVA was used to compare multiple groups across feed height and flow rate, followed by Tukey's pairwise multiple comparison post-hoc test for yield and Tukey's simple effect multiple comparison post-hoc test for size, PDI and zeta potential. Multiple groups across needle diameter were evaluated by one-way ANOVA, followed by Tukey's multiple comparison post-hoc test for size, PDI and zeta potential and the Brown-Forsythe and Welch test, followed by Dunnett's T3 post-hoc test for zeta potential. Error bars are hidden in the bars and plot symbols when not visible, ± SD, $n = 3$. Asterisks denote statistical significance determined using post-hoc tests as follows: *$p < 0.05$, **$p < 0.01$, ***$p < 0.001$, ****$p < 0.0001$. Scale bars 200 nm.

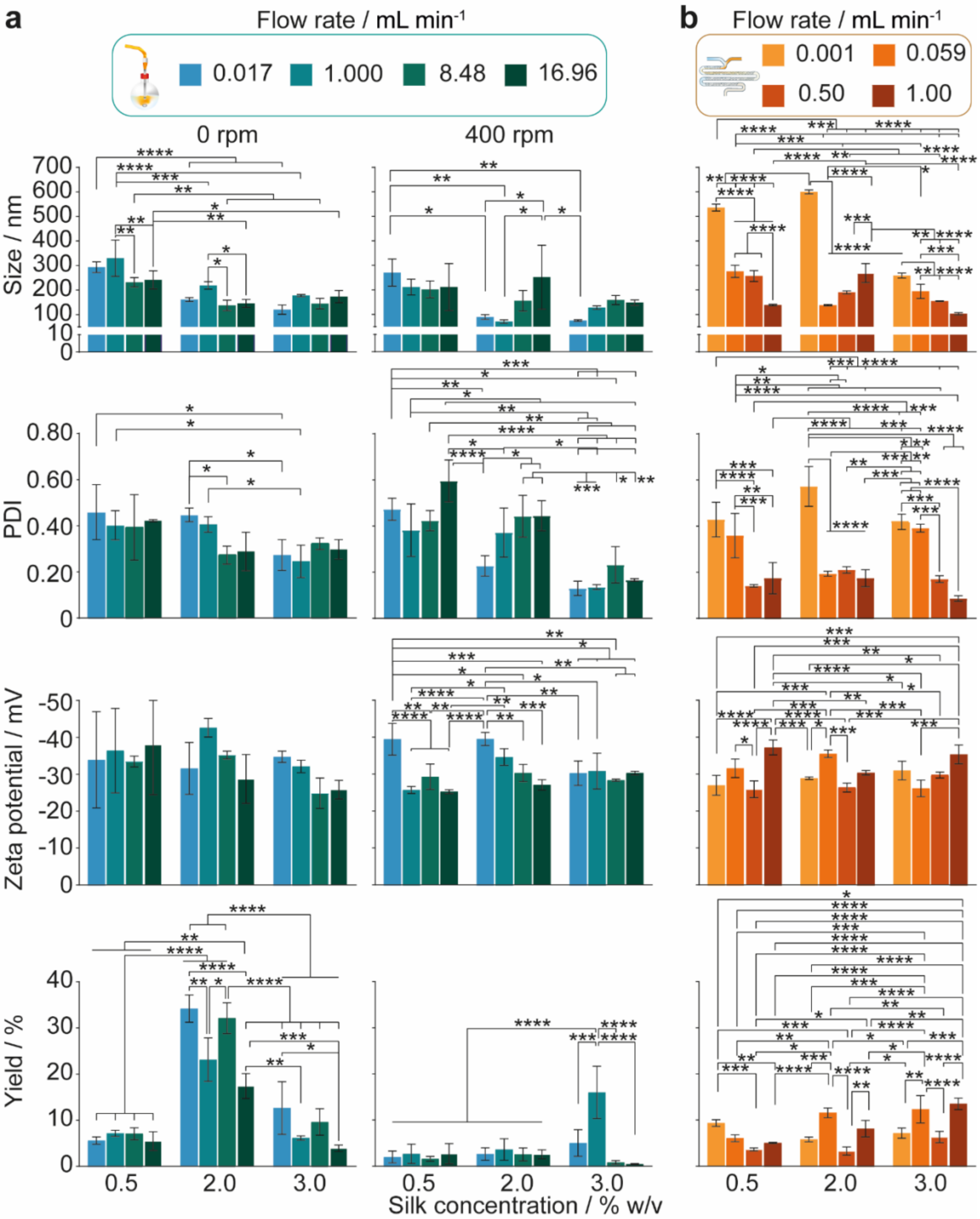

Figure S3. **The impact of increasing the flow rate and silk feed concentration in the staggered herringbone micromixer and in semi-batch systems of high and low mixing time. The hydrodynamic diameter, polydispersity index, zeta potential and yield for (a) semi-batch format and (b) microfluidic format.** For the unstirred semi-batch processes, two-way ANOVA was used to compare multiple groups across concentration and flow rate, followed by Tukey's pairwise multiple comparison post-hoc test for yield and Tukey's simple effect multiple comparison post-hoc test for size, polydispersity index and zeta potential. In stirred semi-batch format and microfluidic format, multiple groups were evaluated by two-way ANOVA, followed by Tukey's pairwise multiple comparison post-hoc test for all properties. Error bars are hidden in the bars when not visible, ± SD, n = 3. Asterisks denote statistical significance determined using post-hoc tests as follows: *p < 0.05, **p < 0.01, ***p <0.001, ****p < 0.0001.

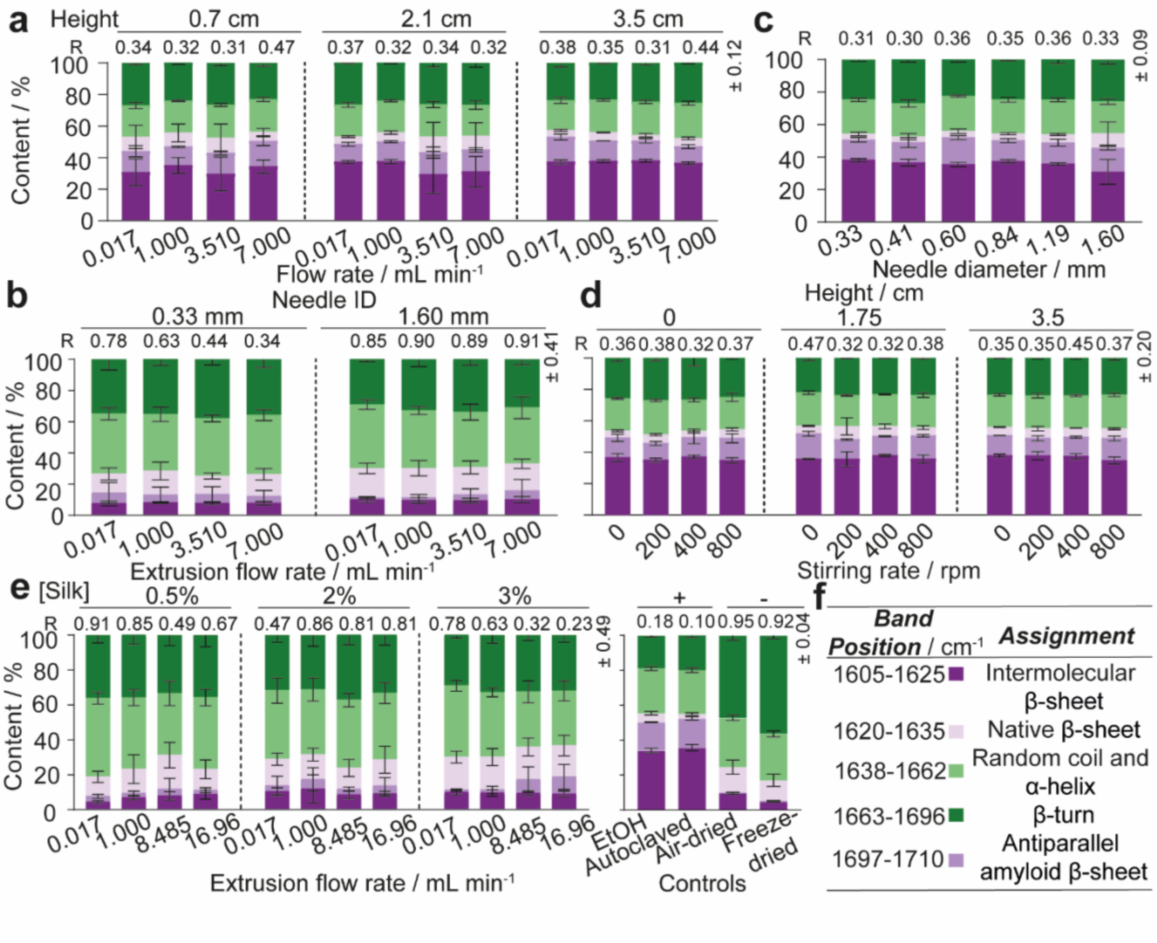

Figure S4. The secondary structure of silk nanoparticles manufactured at the specified formulation parameters in the semi-batch closed system at (a, c) 0 rpm, and (d) with varying initial addition height and stirring rate and (b,e) silk extruded from the closed system feed needles at the specified formulation parameters. (f) FTIR band assignments key. Silk films treated with 70 % ethanol and autoclaving to obtain high β-sheet content were used as positive controls for silk II structure, with an untreated silk film and freeze-dried silk powder serving as negative controls. Two-way ANOVA was used to compare multiple groups across feed height and flow rate (± SD, n = 3), followed by Tukey's pairwise multiple comparison post-hoc test for total β-sheet and anti-parallel amyloid β-sheet contents and Tukey's simple effect multiple comparison post-hoc test for β-turn, intermolecular β-sheet, native β-sheet and α-helix and random coil contents and correlation coefficients. Multiple groups across needle diameter (± SD, n = 3) were evaluated by one-way ANOVA, followed by Tukey's multiple comparison post-hoc test for total β-sheet, anti-parallel amyloid β-sheet, β-turn, α-helix and random coil contents and correlation coefficients while the intermolecular β-sheet and native β-sheet contents were evaluated by the Brown-Forsythe and Welch test, followed by Dunnett's T3 post-hoc test. Two-way ANOVA was used to compare multiple groups across feed height and stirring rate, followed by Tukey's simple effect multiple comparison post-hoc test for the correlation coefficients and contents. Two-way ANOVA was used to compare multiple groups across flow rate and needle diameter (± SD, n = 9), followed by Tukey's pairwise multiple comparison post-hoc test for correlation coefficients and Tukey's simple effect multiple comparison post-hoc test for total β-sheet contents. Two-way ANOVA was used to compare multiple groups across flow rate and concentration (± SD, n = 9), followed by Tukey's pairwise multiple comparison post-hoc test for total β-sheet contents and correlation coefficients. Error bars are hidden in the bars and plot symbols when not visible. Data obtained from silk II controls have been published elsewhere.[2] ± SD, n = 3.

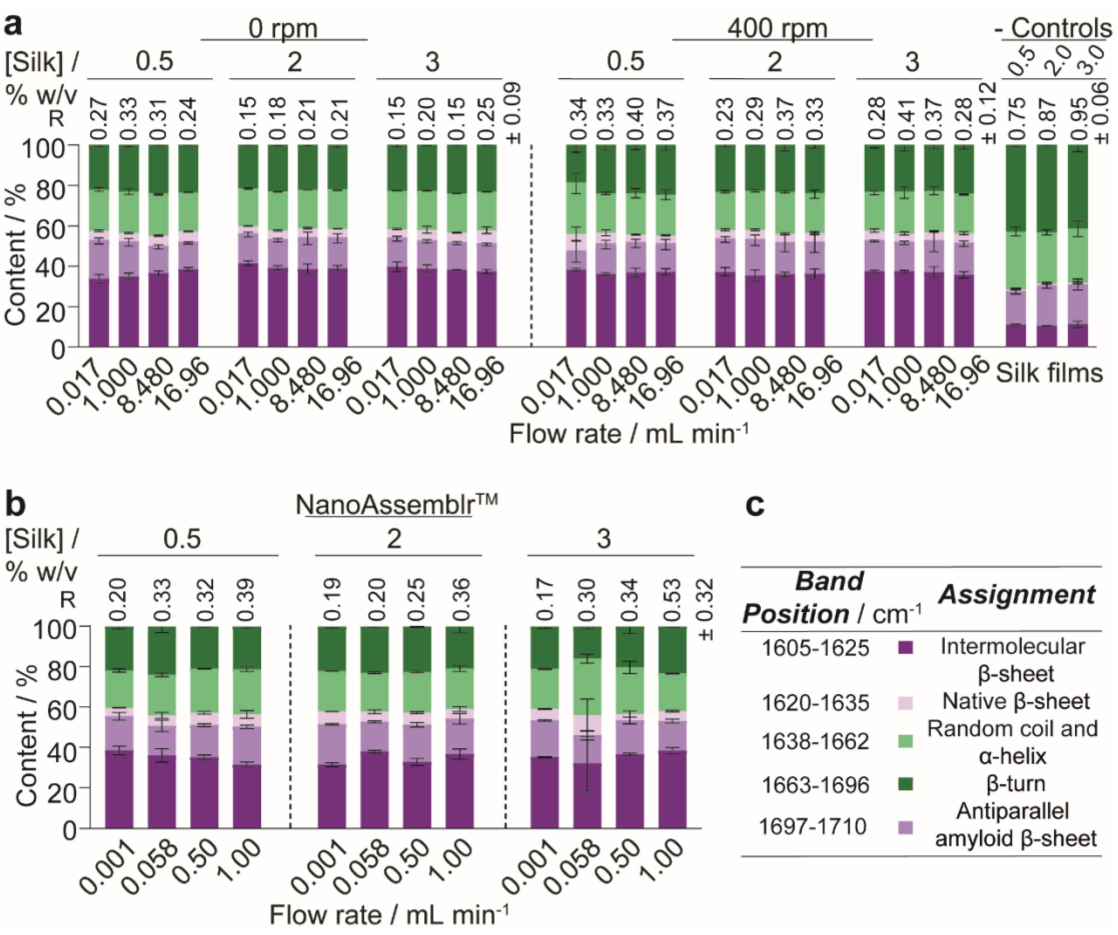

Figure S5. Formulation parameters had a significant impact on the secondary structure of silk nanoparticles. Secondary structure content (%) of silk nanoparticles manufactured using different silk concentrations and flow rates in the **(a)** low mixing time (400 rpm) and high mixing time (0 rpm) semi-batch processes, and **(b)** in the NanoAssemblr™. **(c)** FTIR band assignments key. Untreated silk films drop-casted using 0.5, 2 and 3% silk stocks served as negative silk II controls. Secondary structure content (%) was calculated from the relative areas of peaks in the second derivative spectrum. The correlation coefficients of silk nanoparticle second derivative amide I spectra were calculated using the silk II negative control film as reference. Two-way ANOVA was used to compare multiple groups across concentration and flow rate. In the stirred system, this was followed by Tukey's simple effect multiple comparison post-hoc test for correlation coefficients and the contents for total β-sheet, antiparallel β-sheet, intermolecular β-sheet, random coil and α-helix and β-turn. Native β-sheet content was evaluated using the Tukey pairwise multiple comparison post hoc test. In the unstirred system, total β-sheet, antiparallel β-sheet, intermolecular β-sheet, and β-turn content were evaluated using the Tukey pairwise multiple comparison post hoc test while the Tukey simple effect post hoc test was used for correlation coefficients and the contents for random coil and α-helix and native β-sheets. For the micromixer, correlation coefficients and contents were evaluated using the Tukey simple effects post hoc test except for β-turn and random coil and α-helix content, for which the Tukey pairwise comparison post hoc test was used. ± SD, n = 3.

References

1 X. Jiang, L. Zheng, H. Wu and J. Zhang, *MBE*, 2021, **18**, 4071–4083.

2 S. A. L. Matthew, J. D. Totten, S. Phuagkhaopong, G. Egan, K. Witte, Y. Perrie and F. P. Seib, *ACS Biomaterials Science and Engineering*, 2020, **6**, 6748–6759.
